# 3D genome architecture coordinates *trans* and *cis* regulation of differentially expressed ear and tassel genes in maize

**DOI:** 10.1186/s13059-020-02063-7

**Published:** 2020-06-16

**Authors:** Yonghao Sun, Liang Dong, Ying Zhang, Da Lin, Weize Xu, Changxiong Ke, Linqian Han, Lulu Deng, Guoliang Li, David Jackson, Xingwang Li, Fang Yang

**Affiliations:** 1 National Key Laboratory of Crop Genetic Improvement, Huazhong Agricultural University, Wuhan, 430070 People’s Republic of China; 2 State Key Laboratory of Agricultural Microbiology, Huazhong Agricultural University, Wuhan, People’s Republic of China; 3 Cold Spring Harbor Laboratory, Cold Spring Harbor, NY 11724 USA

**Keywords:** Maize inflorescence, 3D genome, Epigenetic modifications, *Trans* and *cis* regulation, Differential expression, GWAS

## Abstract

**Background:**

Maize ears and tassels are two separate types of inflorescence which are initiated by similar developmental processes but gradually develop distinct architectures. However, coordinated *trans* and *cis* regulation of differentially expressed genes determining ear and tassel architecture within the 3D genome context is largely unknown.

**Results:**

We identify 56,055 and 52,633 open chromatin regions (OCRs) in developing maize ear and tassel primordia using ATAC-seq and characterize combinatorial epigenome features around these OCRs using ChIP-seq, Bisulfite-seq, and RNA-seq datasets. Our integrative analysis of coordinated epigenetic modification and transcription factor binding to OCRs highlights the *cis* and *trans* regulation of differentially expressed genes in ear and tassel controlling inflorescence architecture. We further systematically map chromatin interactions at high-resolution in corresponding tissues using in situ digestion-ligation-only Hi-C (DLO Hi-C). The extensive chromatin loops connecting OCRs and genes provide a 3D view on *cis-* and *trans*-regulatory modules responsible for ear- and tassel-specific gene expression. We find that intergenic SNPs tend to locate in distal OCRs, and our chromatin interaction maps provide a potential mechanism for trait-associated intergenic SNPs that may contribute to phenotypic variation by influencing target gene expression through chromatin loops.

**Conclusions:**

Our comprehensive epigenome annotations and 3D genome maps serve as valuable resource and provide a deep understanding of the complex regulatory mechanisms of genes underlying developmental and morphological diversities between maize ear and tassel.

## Background

In eukaryotes, open chromatin regions (OCRs) along the genome with depleted nucleosomes harbor various categories of *cis*-regulatory elements (CREs), including promoters, enhancers, and insulators, and are accessible to different *trans*-acting factors, such as transcription factors (TFs) that dynamically modulate gene expression [1–7]. Using high-throughput open chromatin mapping technologies, extensive local CREs proximal to genes and distal CREs in intergenic regions have been identified in mammals and plants [1, 4, 6, 8–12]. Different CREs exhibit distinct epigenetic marks, including histone posttranslational modifications (PTMs), DNA methylation, and chromatin accessibility. For example, mammalian enhancers are frequently marked by H3K27ac and H3K4me1 [13, 14]. Recent advances in studies of 3D genome organization found that regulatory elements, including enhancers, promoters, and insulators, frequently form chromatin loops and affect the expression of associated genes [15–18]. To elucidate genome-wide *cis*-element impacts on transcriptional regulation, extensive high-resolution chromatin maps have been obtained using Hi-C and chromatin interaction analysis with paired-end tag sequencing (ChIA-PET) technologies in animals [16, 17, 19]. In recent years, higher-order genome structures for *Arabidopsis*, rice, maize, and other plants have also been obtained via Hi-C technology [20–25]; however, comprehensive high-resolution chromatin maps involving regulatory elements are still deficient, due to the limited resolution of Hi-C maps. Recent studies of long-range regulatory element interactions in maize seedlings using ChIA-PET technology greatly improve the resolution and reveal the important roles of chromatin loops in gene expression and phenotypic variation [10, 26]. Gene pairs with loops between promoter proximal regions in maize tend to be transcriptionally coordinated, indicating they might be involved in the same developmental process [10, 26].

Maize (*Zea mays*) is a major crop worldwide with two types of inflorescence, the tassel and ear, which initiate by similar developmental processes but develop distinct architectures. Tassel and ear primordia originate from shoot apical meristems and axillary meristems, respectively [27]. They are both made by inflorescence meristems (IMs), which initiate spikelet pair meristems (SPMs) that develop into spikelet meristems (SMs) then floret meristems (FMs). Tassel primordia additionally develop several branch meristems (BMs) from the base resulting in their distinct architecture. At the floral organ stage, the tassel and ear become male and female inflorescences as a result of the degeneration of gynoecium primordia in the tassel florets and stamen primordia in the ear florets, respectively [28–31]. Several genes controlling the inflorescence architecture and sex determination in maize have been characterized [32–38]. *TASSEL BRANCHED 1* (*TB1*) is a well-known domestication gene that suppresses vegetative branching (tillers) [39–41]; *tb1* mutants develop more elongated tillers tipped with a male inflorescence, due to a lack of suppression of axillary meristems and of stamens. *TB1* is more strongly expressed in stamen primordia of ear florets than in tassel florets and may suppress outgrowth of the ear. *TASSELSEED1* and *2* (*TS1* and *TS2*) encode hydroxysteroid dehydrogenases that participate in JA biosynthesis, and produce a cell death signal for pistil abortion in tassel florets [31, 32, 35]. An upstream regulator, *SILKLESS1* (*SK1*), is expressed specifically in upper florets of the ear, where it protects pistils from *TS1*- and *TS2*-mediated elimination signals [38, 42]. Therefore, the temporally and spatially specific expression of related genes contributes to the morphological difference and sex determination in ear and tassel [29, 31, 32, 35, 38, 40]. In addition, GWASs found that a large proportion of genetic variants reside in CREs and are associated with ear- and tassel-related agronomic trait variation [43–48]. However, how these CREs exert control on the differential expression of key genes is largely unknown.

In this study, we identified extensive local and distal OCRs in ear and tassel, respectively, and characterized their epigenetic features by investigating genome-wide chromatin accessibility, DNA methylation, and three types of histone modification. The dynamic chromatin states of OCRs affected TF access and were significantly associated with gene expression, which help explain the differences in ear and tassel architectures. We also generated high-resolution chromatin interaction maps using in situ digestion-ligation-only Hi-C (DLO Hi-C) method [49]. A large number of chromatin loops were detected in immature ear and tassel, and the genome topology and the dynamic epigenetic states of these loops were further investigated. Potential *trans*- and *cis*-regulatory modules regulating ear- and tassel-enriched gene expression were associated with loops connecting OCRs and genes. We also found that many intergenic SNPs identified in GWAS for ear and tassel agronomic traits are in chromatin loops. Our results highlight how chromatin loops and epigenetic states of OCRs regulate gene expression to influence the different architectures and identities of the maize ear and tassel.

## Results

### Mapping OCRs and epigenome marks in young ear and tassel

**Fig. 1:**
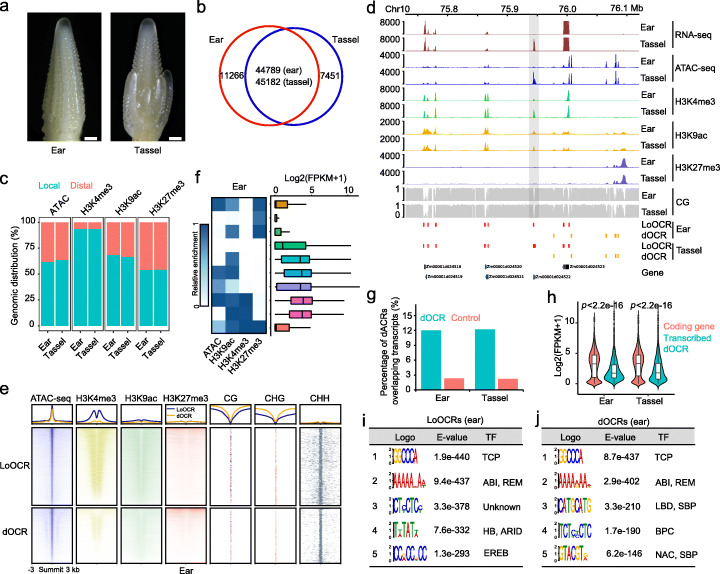
Characterization of OCRs and epigenome marks in maize ear and tassel. **a** Developing ears and tassels (2–4 mm) were dissected for all experiments. Bar = 0.3 mm. **b** A large proportion (80–85%) of OCRs overlapped between ear and tassel, but there were also some tissue-specific OCRs. **c** Distribution of local and distal OCRs and 3 types of histone modification (H3K4me3, H3K9ac, and H3K27me3) in ear and tassel. **d** Gene expression (RNA-seq), chromatin accessibility (ATAC-seq), H3K4me3, H3K9ac, H3K27me3, and DNA methylation (CG) profiles on a selected region of chromosome 10 (75.75–76.1 Mb). LoOCRs and dOCRs in ear and tassel are indicated by red and yellow bars at the bottom, respectively. Gray shading shows an example of dynamic chromatin accessibility and histone modifications with the altered transcription of a nearby gene between ear and tassel. **e** Epigenome profiles at LoOCRs and dOCRs centered on OCR summits in ear. Shown are regions ± 3 kb from OCR summits. **f** The correlation between gene expression levels and different clusters of chromatin accessibility and histone modifications in ear. **g** The percentages of dOCRs that overlap intergenic transcripts in the same tissue in ear and tassel showing that ~ 12% of dOCRs are transcribed. Control: the same number of distal regions as dOCRs generated by randomly shifting dOCRs. **h** Comparison of transcription levels showing that coding genes have a higher expression level than transcribed dOCRs. The Wilcoxon test was used to test significance. **i**, **j** DNA motifs enriched in LoOCRs (**i**) and dOCRs (**j**) of ear. The corresponding candidate motif-binding TFs are shown

Protein *trans* factors bind CREs in OCRs and regulate gene expression [1, 2, 50]. To explore CREs related to differentially expressed genes during ear and tassel differentiation, we first identified OCRs in 2–4 mm ear and tassel primordia of maize (Fig. 1a) using ATAC-seq. The datasets generated from 2 replicates were highly reproducible (Additional file 1: Figure S1a, Additional file 2: Table S1), and 56,055 and 52,633 OCRs were identified in ear and tassel, respectively, including many tissue-specific OCRs (Fig. 1b, Additional file 2: Table S2 and S3). Approximately 63% of the OCRs were close to genes and were defined as local OCRs (LoOCRs: the genomic regions from 3 kb upstream of transcription start sites (TSS), spanning the gene body, to transcription termination sites (TTS) of genes). The remaining OCRs (~ 37%) were located outside the genic regions and defined as distal OCRs (dOCRs) which might be involved in long-range chromatin interactions for transcriptional regulation (Fig. 1c, d). Given the activity of CREs in mammals is associated with certain epigenetic features [13, 51, 52], we investigated the chromatin signatures flanking LoOCRs and dOCRs in ear and tassel, by generating global maps of 3 types of histone modification (active H3K4me3 and H3K9ac and repressive H3K27me3), as well as DNA methylation (Fig. 1d, Additional file 2: Table S2). The analyses of these highly reproducible (*Pearson’s correlation coefficients* from 0.92 to 0.97) epigenome datasets showed that both H3K4me3 and H3K9ac modifications at dOCRs were weaker than those at LoOCRs in both ear and tassel (Fig. 1e, Additional file 1: Figure S1a and S2a). In addition, we found H3K9ac, an enhancer flag in mammals, was depleted in many dOCRs (Fig. 1e, Additional file 1: Figure S2a). In the case of H3K27me3 modification, a low enrichment was present in both LoOCRs and dOCRs, but some exceptional OCRs with strong enrichment of H3K27me3 were also observed (Fig. 1e, Additional file 1: Figure S2a). We also found that the majority of LoOCRs and dOCRs had low DNA methylation, supporting their predicted regulatory functions (Fig. 1e, Additional file 1: Figure S2a). To understand the significance of these chromatin features, we compared them to gene expression levels and found that genes that associated with local chromatin accessibility and active histone modifications (H3K4me3 and H3K9ac) were significantly more active than those with repressive H3K27me3 or lacking all modifications (Fig. 1f, Additional file 1: Figure S2b). We also found that dOCRs tended to overlap with expressed intergenic transcripts, which might represent putative enhancer RNAs (Fig. 1g), and the level of dOCR-associated transcripts was lower than that of protein-coding genes, consistent with the attribute of non-coding RNAs (Fig. 1h, *p* < 2e−16, Wilcoxon test). We next asked about the potential information content of OCRs and found that many putative TF binding motifs were enriched in LoOCRs and dOCRs, such as predicted TCP, LBD, SBP, HB, and EREB binding motifs (Fig. 1i, j, Additional file 1: Figure S2c, d) [53, 54], suggesting that OCRs containing potential CREs recruit different TFs to regulate gene expression. These results highlight the combinatorial effects of OCRs with various epigenetic features on transcriptional regulation in developing maize ears and tassels.

### Dynamic local OCRs and histone modifications contribute to differential expression of genes between ear and tassel

**Fig. 2:**
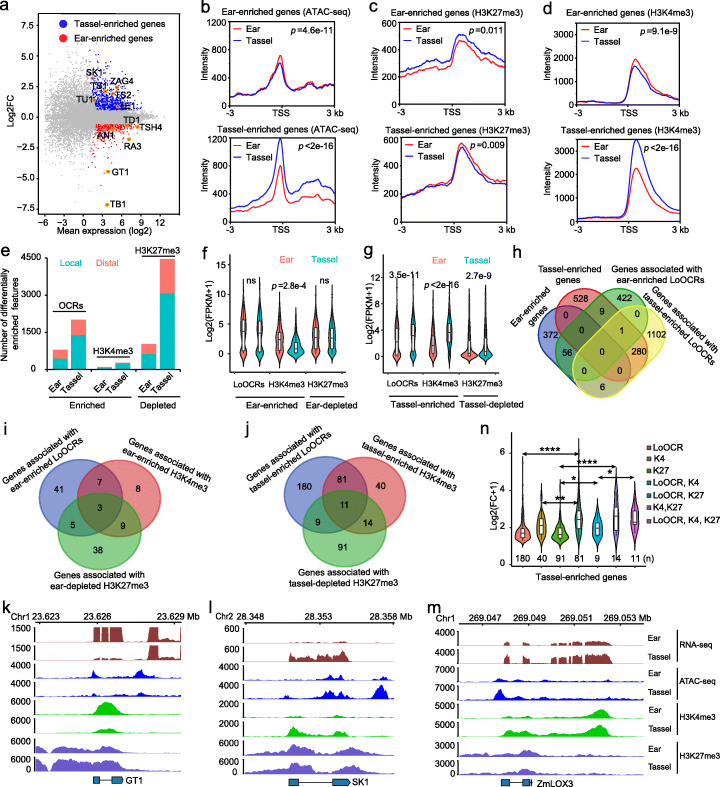
Dynamic LoOCRs and histone modifications associated with differences in gene expression between ear and tassel. **a** MA plot showing differentially expressed genes between ear and tassel, with some known genes marked (highlighted dots with orange circles). Blue dots: 818 tassel-enriched genes; red dots: 434 ear-enriched genes. DEGs were defined with the following criteria: *q* value < 0.01, FC (fold change) > 1.5. **b**–**d** Average signal intensities of ATAC-seq (**b**), H3K27me3 (**c**), and H3K4me3 (**d**) ± 3 kb around the transcription start sites (TSSs) of ear-enriched genes (upper panel) and tassel-enriched genes (lower panel). Ear-enriched genes showed more open chromatin states, stronger H3K4me3, and weaker H3K27me3 modifications in ear, and tassel-enriched genes showed the same results in tassel. The Wilcoxon test was used to test significance. **e** Number of differentially enriched local and distal OCRs, H3K4me3, and H3K27me3 modifications in ear and tassel. **f**, **g** Expression levels of genes with ear-enriched LoOCRs and H3K4me3 and ear-depleted H3K27me3 modifications in ear (**f**) and genes with tassel-enriched LoOCRs and H3K4me3 and tassel-depleted H3K27me3 modifications in tassel (**g**). The Wilcoxon test was used to test significance. ns—*p* > 0.05. **h** Venn diagram showing the overlap between tissue-enriched genes and genes associated with tissue-enriched LoOCRs. **i**, **j** Numbers of ear-enriched (**i**) and tassel-enriched (**j**) genes associated with different chromatin features that are differentially present between ear and tassel. **k**–**m** Examples of an ear-enriched gene, *GT1* (branch suppression) (**k**), and tassel-enriched genes, *SK1* (sex determination) (**l**) and *ZmLOX3* (JA biosynthesis) (**m**), showing differences in their expression and chromatin states in ear and tassel. **n** FC (fold change) in the expression of tassel-enriched gene sets associated with single or combined chromatin state changes. The numbers of gene in different gene sets are shown at the bottom. K4, H3K4me3; K27, H3K27me3. *p < 0.05, **p < 0.01, ****p < 0.0001

**Fig. 3:**
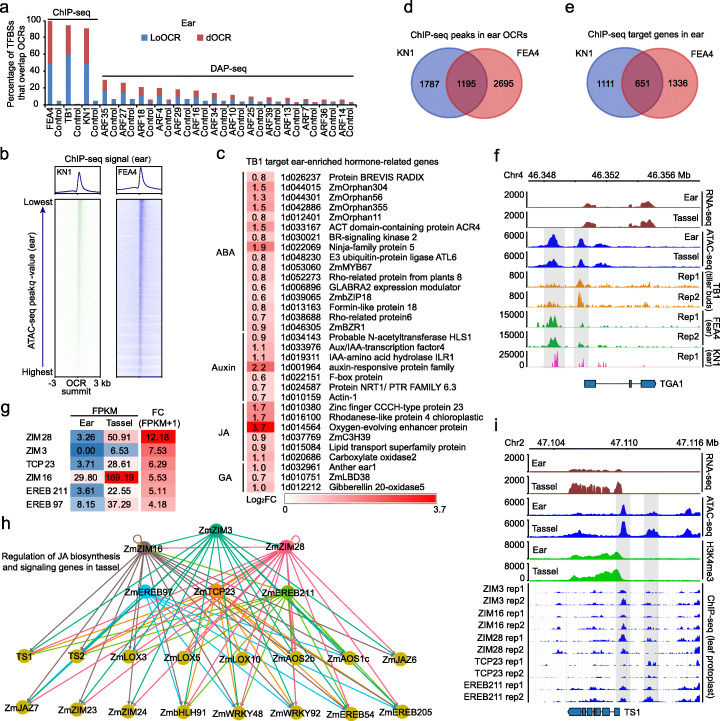
Dynamic TF binding to LoOCRs contributes to differential gene expression between ear and tassel. **a** Percentages of TF binding sites (TFBSs) for 3 TFs identified by in vivo ChIP-seq and TF binding sites for 14 TFs identified by in vitro DAP-seq that overlap with LoOCRs and dOCRs in ear. Controls: the same number of local and distal regions as LoOCRs and dOCRs, respectively, generated by randomly shifting dOCRs. **b** Distribution of the intensity of TF binding centered on ATAC-seq peak summits ordered from bottom to top by descending peak *q* value in ear. Shown are the regions ± 3 kb from peak summits. **c** Ear-enriched genes that respond to ABA, auxin, JA, and GA targeted by ear-enriched expressed TB1 identified by in vivo ChIP-seq. FC, fold change. The color scale indicates the log of fold changes in expression (log2FC). **d**, **e** Overlaps of binding sites of KN1 and FEA4 on OCRs (**d**) and their bound genes (**e**) in ear. **f** The OCRs near *TGA1* overlapped with the potential binding sites of *TB1*, *FEA4*, and *KN1*. **g** Differential expression levels (FPKM) of 6 tassel-enriched TFs in ear and tassel. FCs (fold changes of “FPKM + 1”) are shown in ascending order in the right-hand column in the heatmap. **h** A proposed network of 6 tassel-enriched TFs (top two rows) and their potentially regulated genes (bottom two rows) involved in JA biosynthesis and response. These regulated genes are tassel-enriched and show more open chromatin states in tassel than in ear, except for *TS2*, *ZmLOX5*, and *ZmAOS1c*. *ZmZIM16* and *ZmZIM28* show self-binding. **i** A tassel-enriched gene *TS1* (sex determination) shows higher chromatin accessibility and more H3K4me3 modifications in tassel than in ear. Its LoOCRs overlapped with the binding sites of 5 tassel-enriched TFs (ChIP-seq panel)

The discrepancy in architectures between ear and tassel is likely due to transcriptional differences. We identified 434 ear-enriched and 818 tassel-enriched genes from our generated reproducible RNA-seq datasets (FPKM fold change > 1.5, *q* value < 0.01) (Fig. 2a, Additional file 1: Figure S1b, Additional file 2: Table S1 and S4). Some key genes for ear development, including *TB1*, *GRASSY TILLERS1* (*GT1*), and *ANTHER EAR1* (*AN1*) [33, 36, 55], were among these ear-enriched genes, while tassel-enriched genes included *SK1*, *TS1*, and *TS2* [32, 35, 38]. The differentially expressed genes (DEGs) between ear and tassel primordia were enriched in pathways responding to oxygen-containing compound, hormone, and transcription factor activity by gene ontology (GO) analysis using agrigo [56] (Additional file 1: Figure S3a, b). Particularly, ear-enriched genes were enriched in pathway of gibberellin (GA), which positively controls ear development [55, 57, 58], and tassel-enriched genes were enriched in pathway of jasmonic acid (JA), which mediated pistil abortion in tassels [32, 35, 59, 60]. We further explored the effects of chromatin accessibility and histone modifications on differential gene expression in ear and tassel and found that the ear- and tassel-enriched genes followed similar trends, as they both exhibited more open chromatin and active H3K4me3 modifications and fewer repressive H3K27me3 modifications (*p* < 0.01, Wilcoxon test) (Fig. 2b–d). Next, we analyzed differential peaks of ATAC-seq and histone ChIP-seq using DiffBind [61] to identify differential enrichments of OCRs, H3K4me3, and H3K27me3 between ear and tassel, including differential local and distal enrichments (Fig. 2e, Additional file 2: Table S5, S6 and S7). Genes nearby these differential local enrichments exhibited different expression levels (Fig. 2f, g). Genes associated with tassel-enriched LoOCRs and H3K4me3 modifications and tassel-depleted H3K27me3 modifications were more highly expressed in tassel than in ear (*p* < 2.7e−9, Wilcoxon test) (Fig. 2g), and genes associated with ear-enriched H3K4me3 modifications were more highly expressed in ear than in tassel (*p* = 2.8e−4, Wilcoxon test) (Fig. 2f). The ear-enriched LoOCRs and ear-depleted H3K27me3 modifications overlapped more ear-enriched genes than tassel-enriched genes, although the overall expression levels of genes associated were not observed significantly different between ear and tassel (Fig. 2f, h, Additional file 1: Figure S3c, d). Overall, 111 (26%) of the ear-enriched genes and 426 (52%) of the tassel-enriched genes were found to be correlated with changes in at least one type of chromatin feature (Fig. 2i, j, Additional file 2: Table S4), including some inflorescence development, sex determination, and hormone-related genes, such as *GT1*, *SK1*, *lipoxygenase3* (*ZmLOX3*), *TB1*, *AN1*, *TS1*, and *TS2* [32, 35, 36, 38, 40, 55] (Fig. 2k–m and Fig. 3i, Additional file 1: Figure S3e-h). We observed that combined chromatin feature changes have greater impacts on the expression dynamics of associated genes than a single chromatin feature change, suggesting that gene expression could be fine-tuned by the coordination of multiple chromatin features (Fig. 2n). Taken together, our results demonstrate that the dynamics of local chromatin accessibility and histone modifications are correlated to differential expression of genes that may play important roles in the development and identity of ear and tassel.

### Dynamic TF binding to LoOCRs drives differential gene expression between ear and tassel

The OCRs with appropriate chromatin structure and environment serve as docks that allow TFs to bind specific DNA sequences and regulate gene transcription [62, 63]. A large number of TFs were found differentially expressed between ear and tassel (Additional file 1: Figure S4a). We therefore examined the correlation between OCRs and TF binding sites (TFBSs) using three in vivo ChIP-seq datasets, including KNOTTED 1 (KN1), a meristem maintenance regulator [64]; FASCIATED EAR 4 (FEA4), a regulator of meristem size [65]; and TB1, a regulator of meristem determinacy and an important domestication gene [66]. More than 90% of high-confident binding sites for these 3 TFs overlapped with LoOCRs or dOCRs (Fig. 3a, Additional file 1: Figure S4b), suggesting that these overlapping sites might be the regulatory regions of the three TFs present in ear and tassel. We further analyzed public DAP-seq (DNA affinity purification sequencing) data of AUXIN RESPONSE FACTOR (ARF) TFs [67] and also found more binding sites of TFs overlapping with OCRs than controls, although the proportion was lower than that for the in vivo ChIP binding sites (Fig. 3a, Additional file 1: Figure S4b). In addition, we found that the intensity of open chromatin signals was positively correlated with binding peaks of KN1 and FEA4 in ear (Fig. 3b). TB1, an ear-enriched gene, targeted the LoOCRs of 135 (31%) ear-enriched genes, including hormone response genes, which might contribute to the differential expression of these genes between ear and tassel (Fig. 3c, Additional file 1: Figure S4c, Additional file 2: Table S4). Moreover, we observed 1195 overlapped binding sites of KN1 and FEA4 that occupied 651 common target genes in ear, suggesting they function together in some cases to regulate transcription (Fig. 3d, e, Additional file 2: Table S8). We also found that ~ 30% of the TB1 target genes in tiller buds were overlapped with the targets of KN1 or FEA4. For example, one co-bound gene is *teosinte glume architecture 1* (*tga1*), a domestication gene responsible for the reduced, soft glumes found in modern maize compared to teosinte, previously reported as a putative TB1 target [66, 68], and here, we found it was also bound by FEA4 and KN1 (Fig. 3f).

To investigate the potential regulatory mechanism of tassel-specific TFs, we retrieved the binding sites of six tassel-enriched TFs (3 ZIMs, 2 EREBs, and 1 TCP) from the pre-release cistrome database for 112 TFs’ ChIP-seq in maize leaf protoplast (http://www.epigenome.cuhk.edu.hk/C3C4.html) (Fig. 3g). We found that > 65% of the binding sites of these six TFs overlapped with OCRs in tassel, although the tissues were different. Therefore, we speculated that the TF binding sites overlapping with tassel OCRs were potential regulatory sites of these TFs in tassel and were used for further analysis. The LoOCRs of 411 (~ 50%) of the tassel-enriched genes were found to have at least one binding site of these TFs, which may represent tassel-specific TF regulation relative to ear (Additional file 2: Table S4). Several genes involved in JA biosynthesis and signaling were potentially bound by these 6 TFs, consistent with JA is required for male reproductive identity and suppression of female organ biogenesis [32, 35, 60]. We then constructed a proposed regulatory network comprising of the 6 tassel-enriched TFs and their potential targets that are all tassel upregulated, including JA biosynthesis genes (such as *TS1* and *TS2*, *ZmLOX3*, and *allene oxide synthase2b* (*ZmAOS2b*)), JA response genes (such as jasmonate ZIM-domain protein *ZmJAZ6* and *ZmJAZ7*) [59, 69], and others (Fig. 3h). Except for *TS2*, *ZmLOX5*, and *ZmAOS1c*, the other target genes were also found to have more open chromatin states in tassel than in ear (Additional file 2: Table S4). For example, *TS1*, which had higher chromatin accessibility and H3K4me3 modification in tassel compared to ear, was bound by multiple tassel-specific TFs (Fig. 3i). Interestingly, we found that TFs *ZmZIM16* and *ZmZIM28* could bind their own LoOCRs, suggesting the existence of self-regulatory feedback mechanisms (Fig. 3h). In summary, these results demonstrate that multiple tissue-specific TFs could bind OCRs of target genes and drive their tissue-specific expression to control inflorescence development and determine identity between ear and tassel.

### Mapping chromatin interactions in maize ear and tassel

**Fig. 4:**
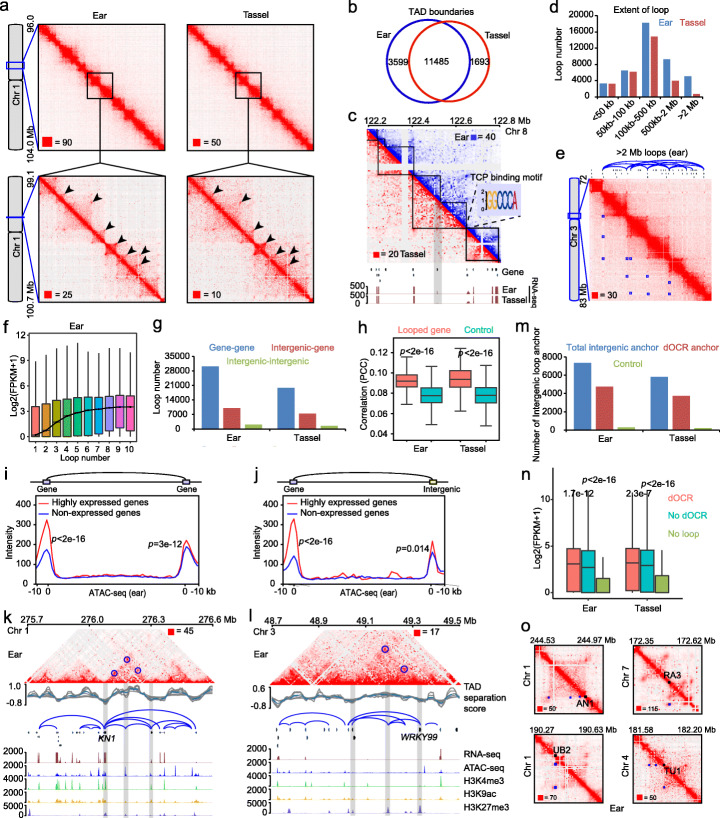
Characterization of TADs and loops and the identification of dOCR-gene loops in ear and tassel. **a** A representative chromatin interaction map of chromosome 1 in ear and tassel. The arrowheads mark several obvious loops. **b** Number of TAD boundaries and tissue-specific TAD boundaries identified in ear and tassel. **c** A case of TAD boundary difference (the boundary is visible only in ear) showing the change in expression of a gene at the boundary (gray shading). The chromatin interaction maps are marked in blue for ear and red for tassel. A DNA motif recognized by TCP TFs is significantly enriched in the TAD boundaries. **d** Number of chromatin loops with different interaction distances in ear and tassel. **e** Obvious chromatin loops (> 2 Mb interaction distance) marked in blue boxes are visible on chromosome 3 in ear. Relevant genes and loop connections are shown above the heatmap. **f** The correlation between gene expression level and the number of loops in ear. **g** Numbers of gene-gene, intergenic-gene, and intergenic-intergenic loops detected in ear and tassel. **h** The expression correlation of gene pairs with real loops is significantly higher than that of randomly selected gene pairs with similar interaction distances (*p* < 2e−16 in both ear and tassel, two-tailed *t* test). PCC, Pearson correlation coefficient. **i**, **j** The average epigenetic features of gene-gene loops showing that highly expressed genes have higher chromatin accessibility together with their looped regions than non-expressed genes in ear (**i**). The intergenic-gene loops have a similar tendency (**j**). Highly expressed genes: FPKM > 100; non-expressed genes: FPKM < 1. The target genes and their looped regions are on the left and right sides of the loops, respectively, in the diagram. Only loops with a distance < 500 kb are included. The Wilcoxon test was used to test significance. **k**, **l** An example of a highly expressed gene, *KN1*, which exhibits active co-modifications with its looped gene and intergenic regions in ear (**k**). In contrast, a non-expressed gene *WRKY99* exhibits repressive co-modifications with its looped gene and intergenic regions in ear (**l**). Gray shadings mark looped regions, and correlated loops in heatmaps are marked with blue circles. **m** Number of total intergenic anchors of loops that overlap with dOCRs in ear and tassel. Control: the same number of distal regions as dOCRs generated by randomly shifting dOCRs. **n** Genes that are looped with dOCRs are significantly more highly expressed than those looped with other regions or without loops. The Wilcoxon test was used to test significance. **o** Examples of four key inflorescence regulation genes involved in chromatin loops (blue boxes) in ear. Gene loci are marked with black dots

Long-range chromatin interactions between CREs and target genes can also have functional implications in transcriptional regulation [16, 17, 19]. We thus adopted the in situ DLO Hi-C method [49] and generated highly reproducible datasets (*Pearson’s correlation coefficients*, 0.90 for ear and 0.93 for tassel) and total 3.2 and 2.2 billion raw read pairs for young ear and tassel, respectively (Fig. 4a, Additional file 1: Figure S5a, Additional file 2: Table S1). After mapping back to the maize genome, we obtained 748 and 493 million valid interactive pair-end tags (PETs) for downstream analysis. The ratio of intrachromosomal versus interchromosomal PETs, which is generally considered to represent the signal-to-noise ratio, in our Hi-C data was 5.36 ± 2.18 (Additional file 1: Figure S5b, Additional file 2: Table S1), indicating our Hi-C data is of high quality (Fig. 4a). We first determined the resolution of our Hi-C maps and found that 95.7% and 78.3% of the ear and tassel 5-kb bins, respectively, had at least 1000 PETs, indicating our Hi-C maps reached a resolution of ~ 5 kb (Additional file 1: Figure S5c).

We next identified 15,084 and 13,178 topologically associating domains (TADs) at 5 kb resolution in ear and tassel, respectively (Additional file 1: Figure S6a). The TAD boundaries in both ear and tassel displayed an obvious enrichment of highly expressed genes, open chromatin, and active epigenetic marks (H3K4me3 and H3K9ac), while the repressive marks H3K27me3 and DNA methylation were poorly enriched or depleted (Additional file 1: Figure S6b-e). We observed that 23% and 13% of TAD boundaries were tissue-specific for ear and tassel, respectively, and a small number of genes at these tissue-specific TAD boundaries were differentially expressed between ear and tassel (Fig. 4b, c). We also found seven DNA motifs that were significantly enriched at TAD boundaries relative to the background, including a motif recognized by plant-specific TCP TFs (Fig. 4c, Additional file 1: Figure S6f, g and S7, see the “Methods” section) [53, 54]. The presence of these TF binding motifs suggests that active gene expression regulation occurs at TAD boundaries. In summary, we have provided high-resolution chromatin interaction maps for ear and tassel primordia, which can greatly facilitate studies of chromatin organization and transcriptional regulation in maize.

### Characterization of chromatin loops and identification of dOCR-gene connections

We further identified 42,300 and 28,748 chromatin loops in ear and tassel, respectively (Fig. 4a, Additional file 1: Figure S5d, e, Additional file 2: Table S9 and S10). These extensive loops provide an opportunity to study the spatial relationship between CREs and target gene expression. The genomic span of chromatin loops was mostly between 100 and 500 kb (Fig. 4d), though we also observed some loops > 2 Mb in length, which may suggest a super-distant gene regulation mechanism (Fig. 4e). Further analysis found a positive correlation between the number of loops and gene expression level (Fig. 4f, Additional file 1: Figure S8a), consistent with the result of a recent ChIA-PET study in maize [10]. We further classified chromatin loops as gene-gene loops, intergenic-gene loops, and intergenic-intergenic loops (Fig. 4g, Additional file 2: Table S9 and S10). We found that ~ 70% of the chromatin loops were gene-gene loops, suggesting that genes form spatial gene clusters. This is supported by the finding that the expression of gene pairs with chromatin loops in both ear and tassel was more highly correlated than randomly selected gene pairs separated by similar distances (*p* < 2e−16 both in ear and tassel, two-tailed *t* test) (Fig. 4h, Additional file 1: Figure S8b). We also identified 10,037 and 7395 intergenic-gene loops in ear and tassel, respectively (Fig. 4g), which potentially mediate long-range chromatin interactions between intergenic CREs and genes. For both gene-gene and intergenic-gene loops, the anchors tended to be with accessible chromatin (Additional file 1: Figure S8c-f). Interestingly, we found that not only highly expressed genes, but also their looped genes or intergenic regions showed moderately higher chromatin accessibility than that of non-expressed genes (*p* < 0.05, Wilcoxon test) (Fig. 4i, j, Additional file 1: Figure S8g, h). For instance, *KN1*, a key inflorescence meristem regulator [70], together with its long-range interacting gene and intergenic regions, had open chromatin as well as active histone modifications (Fig. 4k). In contrast, another gene *ZmWRKY99*, which was not expressed in ear, had opposite chromatin states to *KN1* (Fig. 4l).

**Fig. 5:**
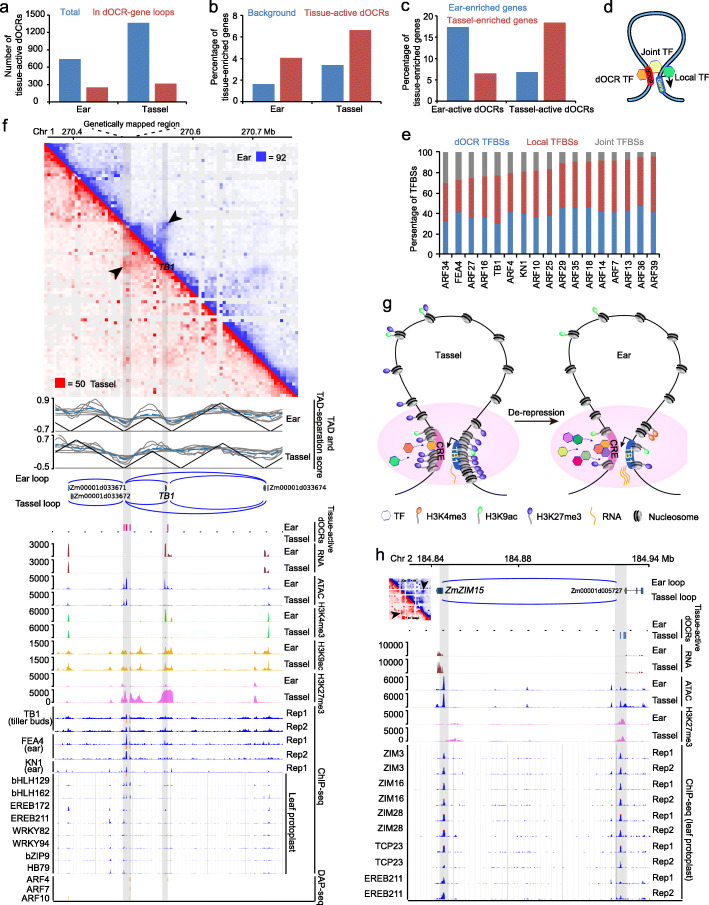
The dynamic activities of TF-bound dOCRs contribute to tissue-specific gene expression. **a** Number of total tissue-active dOCRs and tissue-active dOCRs that are involved in dOCR-gene loops in ear and tassel. **b** The percentages of tissue-enriched genes involved in tissue-active dOCR-gene loops are significantly higher than those of tissue-enriched genes involved in total dOCR-gene loops (background) in ear and tassel. **c** Comparison of the percentages of tissue-enriched genes looped with tissue-active dOCRs shows that ear-active dOCRs tend to interact with ear-enriched genes, while tassel-active dOCRs tend to interact with tassel-enriched genes. **d** A schematic diagram of 3 types of TFs based on their binding to the anchors of dOCR-gene loops. dOCR TF, TF with binding sites on only dOCRs; Local TF, TF with binding sites on only the local OCRs of gene anchors; Joint TF, TF with binding sites on both loop anchors. **e** The percentages of different types of TF binding sites (TFBSs) involved in dOCR-gene loops. dOCR TFBSs, TF binding sites in only the dOCR anchors of dOCR-gene loops; Local TFBSs, TF binding sites in only the gene anchors of dOCR-gene loops; Joint TFBSs, TF binding sites on both anchors of dOCR-gene loops. **f** Example of ear-active dOCRs looping with the *TB1* gene, which is highly expressed in ear but lowly expressed in tassel. Chromatin interaction map (top panel) with obvious loops marked by arrowheads. TAD-separation scores and TADs, chromatin loops, tissue-active dOCRs, RNA levels, epigenetic features, ChIP-seq, and DAP-seq peaks around the dOCRs and *TB1* in ear and tassel are shown. **g** A proposed model for *TB1* regulation showing a de-repression status in ear compared to tassel. The distal *cis*-regulatory element (CRE) of *TB1* is more open with less H3K27me3 modifications, leading to easier access of multiple TFs in ear than that in tassel, which could drive an increased expression of *TB1*. The hexagons with different colors represent different TFs, which might show a sequential and differential binding to CRE in ear and tassel. **h** Case of tassel-active dOCRs looping to a JA response gene, *ZmZIM15*, which has a higher expression in tassel. Chromatin loops, tissue-active dOCRs, RNA levels, epigenetic features, and ChIP-seq data (5 tassel-enriched TFs) around *ZmZIM15* and its dOCRs in ear and tassel are shown. The chromatin interaction map around *ZmZIM15* is shown on the left of the gene track, with loops marked by arrowheads

The extensive dOCRs in maize ear and tassel inspired us to investigate if the dOCRs could interact with their target genes by intergenic-gene loops. Interestingly, we found that more than 60% (4748/7340 in ear, 3740/5827 in tassel) of the intergenic anchors of all intergenic-gene loops contained at least one dOCR (Fig. 4m). These dOCR-gene loops (Additional file 2: Table S11 and S12) included the interaction between *TB1*, *RAP2.7*, and *BX1* and their long-distance regulatory regions [34, 71, 72] (Fig. 5f, Additional file 1: Figure S9). We also found that one dOCR could interact with multiple genes in both ear and tassel (Additional file 1: Figure S10a-d). Interestingly, genes that looped with dOCRs had significantly higher expression level than genes with non-dOCR loops or with no loops (*p* < 2.3e−7, Wilcoxon test) (Fig. 4n), indicating that dOCRs, which likely harbor CREs, are vital for gene regulation. Utilizing our chromatin loop data, we also identified inflorescence development and identity genes that interact with dOCRs or other genes (Fig. 4o, Additional file 1: Figure S10e). For instance, *AN1*, a suppressor of male flower development on pistillate ears [55], was found to interact with one dOCR and two gene regions. *RAMOSA3* (*RA3*), *UNBRANCHED2* (*UB2*), and *TUNICATE1* (*TU1*) that control inflorescence architectures [37, 73, 74] were also found to loop with dOCRs and/or genes (Fig. 4o). Taken together, the identification of dOCR-gene loops, which may provide CRE-gene interaction, provides a view on gene regulation at the 3D level and a new direction for functional studies of key developmental genes.

### Dynamic *trans* and *cis* dOCR regulation contributes to tissue-specific gene expression

Dynamic enhancer landscapes are involved in cell differentiation and organ development processes in a tissue-specific manner to affect target gene transcription in mammals and plants [2, 3, 17, 63]. To ask if dynamic activities of dOCRs affect tissue-specific gene expression, we focused on the dynamic dOCRs, whose accessibility or flanking repressive H3K27me3 modification was significantly different in peaks between ear and tassel. These dynamic dOCRs were divided into ear-active dOCRs (more open and/or weaker H3K27me3 in ear than in tassel, *n* = 738) and tassel-active dOCRs (more open and/or weaker H3K27me3 in tassel than in ear, *n* = 1367). We found that 34% (250/738) and 23% (318/1367) of ear- and tassel-active dOCRs were involved in dOCR-gene loops in ear and tassel, respectively (Fig. 5a, Additional file 2: Table S13 and S14). Next, we found that these ear- and tassel-active dOCRs tended to interact with ear- and tassel-enriched genes, respectively (Fig. 5b, c; Additional file 2: Table S15 and S16). These results indicate that dOCR dynamics contribute to changes in gene expression between ear and tassel. We next examined TF binding events at OCRs within the 3D genome context and observed that 4–30% of TF binding sites involved in dOCR-gene loops were distributed at both anchors of dOCR-gene loops, while the remaining TF binding sites were present either at dOCRs’ anchor or at gene anchor (Fig. 5d, e). This result suggests that transcriptional regulation of genes requires the cooperation of distal and local OCRs to recruit relevant gene expression regulators.

As one case, a genetically mapped distal region (∼ 58–69 kb upstream of the TSS of *TB1*) is known to regulate *TB1* transcription to control branching [34]. *TB1* was more highly expressed in ear than in tassel, which helps elicit its suppression on outgrowth of stamen primordia in ear florets [35]. In our chromatin loop data, we found 3 adjacent dOCRs (~ 59 kb, ~ 64 kb, and ~ 68 kb upstream of the TSS of *TB1*, respectively) overlapping with the genetically mapped distal region of *TB1* (Fig. 5f), and they had higher chromatin accessibility and much lower H3K27me3 modification in ear than those in tassel. Meanwhile, the *TB1* gene region also showed higher open chromatin signals, weaker H3K27me3, and stronger H3K4me3 modifications in ear compared to tassel, suggesting that the dOCRs and gene region are co-modified to regulate *TB1* transcription due to loop-induced spatial proximity. Interestingly, we also observed a difference in TAD boundary location around the *TB1* locus between ear and tassel, with TB1 closer to a TAD boundary in ear than in tassel, in line with the observation that highly expressed genes are enriched at boundaries [22, 75]. Furthermore, we found that the binding sites of multiple TFs (TB1, FEA4, KN1, 3 ARFs, 2 bHLHs, 2 EREBs, 2 WRKYs, bZIP9, and HB79) overlapped with the dOCRs of *TB1*, suggesting that these TFs might bind to the distal *cis* elements of *TB1* and form a regulatory module in ear and/or tassel. Collectively, these results suggest that the chromatin interactions between *TB1* and its dOCRs provide 3D topology for simultaneous de-repression of H3K27me3 at both sites in ear versus tassel, with more open chromatin to facilitate multiple TFs’ access to activate *TB1* transcription in ear (Fig. 5g). In addition, another tassel-enriched JA response gene *ZmZIM15* was found to form a loop with its ~ 82 kb upstream dOCRs, which had more open chromatin and weaker H3K27me3 modification in tassel than in ear (Fig. 5h). Moreover, several tassel-specific TFs were found to potentially bind at both LoOCR and dOCRs, except for *ZmEREB211*, which might be involved in stabilizing chromatin loops to regulate *ZmZIM15* transcription. This result is consistent with the known role of ZIM family TFs in JA responses. Additional genes with differences in long-range interacting dOCRs between ear and tassel include *RA3* [73], *ZmSBP10*, and *SAR DEFICIENT 1* (Additional file 1: Figure S11). Taken together, we propose that dynamic dOCRs may provide variation in chromatin structure and environment, to affect TF access and regulate gene transcription differences between ear and tassel.

### Chromatin loops link trait-associated intergenic SNPs to target genes for potential control of phenotypic variation

**Fig. 6:**
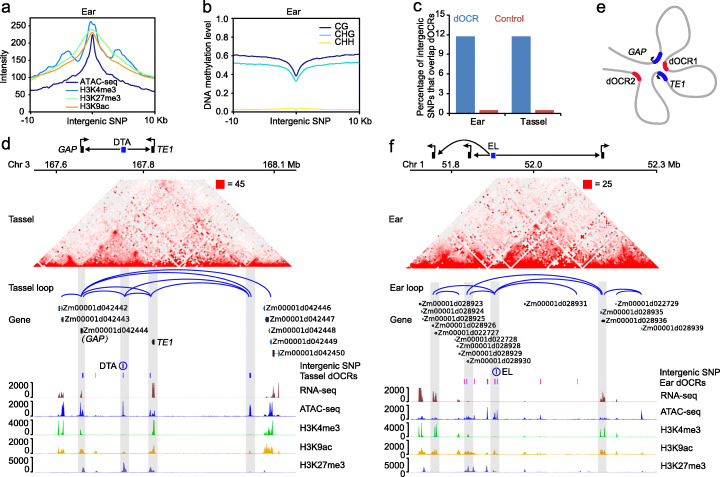
Intergenic SNPs tend to locate in dOCRs and connect with their target genes by intergenic-gene loops. **a**, **b** The average distribution of chromatin accessibility (ATAC-seq), histone modifications (**a**), and DNA methylation (CG, CHG, CHH) levels (**b**) centered at trait-associated distal SNPs in ear. **c** Percentages of intergenic SNPs that overlap with dOCRs. Control uses the same number of distal regions as dOCRs by randomly shifting dOCRs. In total, 49,117 intergenic SNPs were used for the calculation for **a–c**. **d** A flowering time SNP (DTA, days to anthesis) adjacent to one dOCR interacts with two genes, *TE1* and *GAP*, through loops. **e** A proposed model showing the chromatin folding comprising *TE1*, *GAP*, and dOCRs. The *TE1* and *GAP* genes are marked in blue, and dOCRs are marked in red. dOCR1 is closer to *TE1* and *GAP* than dOCR2, based on the stronger chromatin interaction intensities. **f** An ear length (EL) SNP interacting with its multiple potential target genes. Gray shadings mark the genome regions involved in loops, and the blue circles mark trait-associated SNPs (**d**, **f**)

Genome-wide association study (GWAS) has identified many genic and intergenic SNPs associated with agronomic traits in maize [48, 76]. The pervasive long-range chromatin interactions inspired us to explore the functional importance of the intergenic SNPs. We first analyzed the 0.2 million natural SNPs generated from a maize association panel of 513 inbred lines [48] and found that 23% of these SNPs located in intergenic regions, more than 3 kb upstream or downstream of the TSS or TTS, respectively. These intergenic SNPs were found to be enriched in open chromatin regions with strong H3K4me3, H3K9ac, and H3K27me3 modifications and low DNA methylation (Fig. 6a, b, Additional file 1: Figure S12a, b). We further observed that about 11% of intergenic SNPs located in dOCRs, which were significantly higher than that overlapped with randomly selected distal regions (Fig. 6c), suggesting that intergenic SNPs in dOCRs might be functional elements.

To investigate the potential mechanism of intergenic SNPs contributing to agronomic traits, we collected publicly available SNPs that are significantly associated with ear and tassel traits (Additional file 2: Table S17) [76], and found there were 33% (527/1621) SNPs located in distal intergenic regions. Within a 5-kb window centered on trait-associated intergenic SNPs, 135 SNPs are involved in 305 intergenic-gene loops, which suggested that these intergenic SNPs might influence agronomic traits by regulating their potential target genes through loops. As one example, one flowering time SNP for days to anthesis (DTA) immediately adjacent to one dOCR formed chromatin loops with *TERMINAL EAR 1* (*TE1*) and a GTPase activating protein (*GAP*) (Fig. 6d). *te1* mutant is known to have more leaves initiated, a defect that is tightly related to flowering time [77]. We also observed that *TE1* formed loops with a *GAP* gene and another dOCR, which provided a spatial regulatory module related to flowering time (Fig. 6e). In another case, an ear length (EL) SNP that located in dOCR looped with multiple target genes, suggesting these genes may play roles in regulating ear length (Fig. 6f). Taken together, our results suggest that trait-associated intergenic SNPs may influence their target gene expression through long-range chromatin interactions, further contributing to phenotypic variations.

## Discussion

Proper development of ear and tassel is critical for reproduction and grain yield in maize. At an early developmental stage, the ear and tassel have very similar morphologies, but eventually establish different architectures and identities [27]. Exploring the molecular mechanisms underlying their development is not only a key biological question to understand male and female inflorescence architecture and floral organ differentiation, but also paves the way to improve yield traits. Complex gene expression regulatory modules involving *cis*-CREs and *trans*-protein factors are likely to be a driving force during ear and tassel development. Genome-wide identifications of CREs based on open chromatin using DNase I hypersensitive sites (DHSs) and MNase-hypersensitive (MNase HS) regions have been carried out in *Arabidopsis*, rice, cotton, and maize, which greatly promoted the study of plant functional genome [1, 2, 4, 6, 9, 24]. In maize, less than 3% of its genome (coding and MNase HS regions) may give rise to majority of phenotypic variation, greatly narrowing the scope of the functional genome [6]. The degree of chromatin accessibility of CREs and their flanking histone modifications, including active and repressive marks, and DNA methylation level have been used to predict their activities, which can dynamically regulate gene transcription in plants [1, 2, 4, 12]. A subsequently developed technique of assay for transposase-accessible chromatin (ATAC-seq) has been applied in plants, which is more efficient and less tissue-consumed to map open chromatin regions [8, 78–80].

In this study, we identified extensively potential CREs based on open chromatin regions using ATAC-seq and characterized their epigenome features in developing ear and tassel of maize. By integrating hundreds of DEGs between ear and tassel, we provide a valuable resource for understanding the regulation of inflorescence development and sex determination genes, such as *TB1*, *GT1*, *AN1*, *SK1*, and *TS1* [35, 38–40, 55]. In addition, we identified GA-related genes enriched in ear and JA-related genes enriched in tassel, consistent with previous studies that these hormones regulate development of ear and tassel, respectively [35, 55, 58, 59, 69]. We also observed that many of the DEGs were associated with changes of local chromatin accessibility and/or flanking histone modifications. The combined effects of different epigenetic feature changes on differential gene expression were greater than a single feature change. Our results demonstrate that the dynamics of local chromatin accessibility and histone modifications are significantly correlated to the differential expression of genes that play important roles in the morphological differences and identity of ear and tassel.

We identified several TFs with ear or tassel preferential expression, which inspired us to explore genome-wide TF binding sites. Using published ChIP-seq datasets generated from different tissues (developing tassel and/or ear for KN1 and FEA4, tiller buds for TB1, leaf protoplast for 6 tassel-enriched TFs), we found an extensive overlap between TF binding sites and OCRs, suggesting that OCRs could act as a proxy for TF binding. Furthermore, the signal strength of open chromatin correlated with the strength of TF binding, indicating that OCRs can be used to predict TF access. We also found that a considerable number of binding sites overlap between different TFs, suggesting that they can function together to regulate transcription. To be noted, using the ChIP-seq datasets generated from different tissues may underestimate the differences of TF binding patterns in ear and tassel, although we only considered the high-confident TF binding sites overlapping with ear/tassel OCRs for further analysis. In addition, the DNA binding profile of TB1, which is preferentially expressed in ear, gives us valuable information to investigate organ-specific regulatory pathways. To understand tassel-specific regulation, we examined six tassel-enriched TFs from pre-release maize cistrome data (http://plants.ensembl.org/index.html). Interestingly, we found that 6 TF binding sites are overlapping with the LoOCRs of half of the tassel-enriched genes, indicating their key roles in tassel-specific regulation. A regulatory network of JA biosynthesis and responses derived from these 6 tassel-enriched TFs provides a critical view on the mechanism of JA signaling in controlling pistil abortion during tassel development. Taken together, our results demonstrate that TFs tend to bind the LoOCRs of target genes, and suggest these differentially expressed TFs are critical to determine inflorescence development and architecture.

3D genome topology already provides a lot of information to investigate the genome structure and interaction between CREs and genes in animal [16, 17, 19, 81]. The higher-order genome structures for *Arabidopsis*, rice, maize, and other plants have been obtained via Hi-C technology [20–23, 25, 82]. However, high-resolution chromatin maps involving regulatory elements are still deficient, due to the limited resolution of Hi-C maps. Recent studies of long-range regulatory element interactions in maize seedlings using ChIA-PET technology greatly improve the resolution and reveal the important roles of chromatin loops in gene expression and phenotypic variation [10, 26]. In maize, several long-distance regulatory regions for *TB1*, *ZmRap2.7*, *BX1*, *UNBRANCHED 3* (*UB3*), and *ZmCCT9*, which are related to important agronomic traits, have been genetically mapped. These regions likely contain CREs and control their target gene expression [34, 71, 72, 83, 84]. The widespread presence of dOCRs in ear and tassel therefore prompted us to investigate the chromatin interactions between dOCRs and potential target genes. Our high-resolution chromatin interaction maps obtained via in situ DLO Hi-C technology [49] revealed obvious genome topologies typical of chromatin loops. Most of the loops we identified were gene-gene loops, suggesting extensive regulatory regions in the vicinity of genes. We also identified thousands of dOCR-gene loops, consistent with a recent work on long-range *cis*-regulatory elements in the seedling of maize [85]. Interestingly, we found that genes that interacted with dOCRs tended to be more highly expressed, suggesting that these loops contain CREs. The regulatory input of genes could come from either local or distal binding of TFs, and often comes about through cooperation between multiple TFs in the so-called *cis*-regulatory modules [3]. Enhancers and their activities can influence the binding ability of regulatory TFs to regulate stage-specific gene expression, critical for animal and plant development [2, 3, 17]. In our study, we found that dOCR activity correlated with gene expression differences between ear and tassel. TFs can be recruited by dOCRs, or by LoOCRs of the dOCR-gene loops, to regulate transcription. We also observed that both anchors of dOCR-gene loops can be simultaneously bound by the same transcription factors, suggesting that these factors may stabilize the dOCR-gene interactions.

The distal OCR to gene chromatin interactions identified in this study provides a mechanism for cooperative epigenetic modifications and transcription factor binding. In the case of *TB1*, an important domestication gene [34], both the *TB1* and its dOCRs (~ 64 kb upstream) were more open and contained substantially fewer H3K27me3 modifications in ear than in tassel, indicating that these dOCRs and the *TB1* gene may be co-modified due to their loop-driven spatial proximity. Our findings highlight a new mechanism for the enriched TB1 expression in ear. Furthermore, the binding of several TFs, including KN1, FEA4, and potentially bHLHs, EREBs, WRKYs, ARFs, and TB1 itself, to the dOCRs of *TB1* may be involved in the dynamics of epigenetic features. Meanwhile, *TB1* was enriched with H3K4me3 marks in only the ear. Thus, we speculate that the more active epigenetic features at the *TB1* might be the reason for its more open state in ear, which would facilitate TF access and higher *TB1* transcription in ear. In this way, the differential expression of *TB1* can influence development and morphological differences between ear and tassel.

In recent years, several GWASs have identified a large number of ear and tassel trait relevant SNPs [48, 76], with many falling into intergenic regions. However, how these SNPs function to control ear and tassel architectures and yield is largely unknown. Our analysis revealed that 33% of agronomic trait-associated SNPs were in distal intergenic regions, suggesting they may function at a distance through 3D folding or *cis* regulation. Furthermore, some of these intergenic SNPs located in dOCRs, suggesting they may influence the functions of CREs and their target genes. Chromatin loops identified in this study provide valuable information on connections between these trait-associated intergenic SNPs and their potential target genes, important for understanding the regulatory mechanism of these SNPs. As an interesting case, a SNP for flowering time immediately adjacent to one dOCR was found to form a loop with the gene, *TE1*, whose mutation interrupts leaf initiation and leads to form more leaves, a process tightly related to flowering time [80]. Particularly, *TE1* was also found to interact with another dOCR and the gene *GAP* through chromatin loops which provided a spatial regulatory module for a comprehensive understanding of *TE1* and its components on the control of flowering time and the relevant traits. These results demonstrate that trait-associated intergenic SNPs can be linked to target genes to understand their potential roles in phenotypic variation.

In summary, our comprehensive epigenome characterization on distal and local OCRs, and high-resolution 3D genome maps, provides a deep understanding of the control of differential transcription underlying developmental and morphological diversity of the maize ear and tassel. Our genome-wide identification of OCRs aids an underlying CRE annotation to illustrate the dynamic *cis* and *trans* regulation of target genes. Integrated analysis of coordinated epigenetic modifications and TF binding on distal and local OCRs showed that the 3D genome topology can coordinate *trans* and *cis* regulation of differentially expressed genes in ear and tassel. In addition, agronomic trait-associated intergenic SNPs can be connected to potential target genes via loops that may control phenotypic variations. These findings provide insights into ear- and tassel-specific regulatory pathways and can be utilized in crop improvement.

## Conclusions

We generate high-resolution Hi-C maps and characterize epigenome features of OCRs in maize ear and tassel, which are the two types of inflorescences critical for corn yield. Our findings highlight the epigenetic features of OCRs and TFs binding to OCRs responsible for the *cis* and *trans* regulation on DEGs that may control ear and tassel inflorescence architecture and identity. We also identified extensive chromatin loops connecting OCRs and genes that provide a 3D view on *cis*- and *trans*-regulatory modules for ear- and tassel-specific gene expression. We finally propose a potential mechanism for ear and tassel trait-associated intergenic SNPs that may contribute to phenotypic variation by influencing target gene expression through loops. In a general perspective, our comprehensive characterizations on OCRs at epigenetic level, *cis* and *trans* levels, as well as at 3D view would provide a deep understanding of gene differential regulation underlying developmental and morphological diversity in maize.

## Methods

### Materials

Ear and tassel at 2–4 mm in length were harvested from maize inbred line B73 grown in the field. For Hi-C and ChIP-seq, the samples were cross-linked in PBS with 1% formaldehyde for 15 min under vacuum, and the fixation was terminated by adding glycine to 0.2 M for another 5 min under vacuum. Then, cross-linked samples were rinsed thrice with deionized water and immediately frozen in liquid nitrogen and stored at − 80 °C before use. The RNA-seq datasets had 3 replicates, and ATAC-seq, ChIP-seq, and Hi-C datasets had 2 replicates for each tissue. BS-seq had one replicate with sufficient sequencing depth (30×).

### Hi-C library preparation

The Hi-C libraries were constructed according to the published in situ DLO Hi-C protocol with some modifications [49]. For each replicate, about 60 cross-linked ears or tassels were chopped with a razor blade in PP buffer (1× PBS containing 1× protease inhibitor) to obtain the nuclei. After the mixture was filtered through miracloth, SDS was added to the filtrate to a final concentration of 0.5% to permeabilize the nuclei at 65 °C for 5 min. Then, SDS was immediately quenched by adding Triton X-100 to a final concentration of 1% and the mixture was incubated at 37 °C for 10 min. Next, the nuclei were pelleted and washed once with PP buffer, then were incubated in 400 μl MseI digestion buffer with 30 μl MseI restriction enzyme (NEB, R0525L) and 1% Triton X-100 for 8–10 h at 37 °C with rotation at 15 rpm. After digestion, MseI half-linkers (forward strand: 5′-[5Phos]TAGTCGGAGAACCAG/iBiodT/AG-3′, reverse strand: 5′-CTAGCTACTG GTTCTCCGAC-3′) were ligated to the digestion fragment ends using T4 DNA ligase (Thermo, EL0012). After half-linker ligation, the nuclei were pelleted by centrifugation and washed two times with 1.5 ml PT buffer (1× PBS containing 0.5% Triton X-100). Next, the fragment-end phosphorylation, in situ proximity ligation, reversal of cross-linking, and DNA purification were performed as described [49]. The purified DNA fragments which contained the linker at ligation junctions were captured by Dynabeads™ M-280 Streptavidin (Thermo, 60210) and used to construct sequencing library based on Tn5 transposase (VAHTS, TD501). The final amplicons were size-selected using DNA clean beads (KAPA) and submitted for paired-end sequencing (2 × 150 bp) using Illumina Hiseq X-Ten (Annoroad Biotechnology).

### Hi-C data analysis

B73 AGPv4 reference genome was used to analyze all the data. For Hi-C, we filtered linkers and mapped the sequences against the maize genome using ChIA-PET2 [86]. Unmapped reads, low-quality mapped reads (mapping quality scores < 10), and PCR duplicates were removed. Next, the DLO-HiC-Tools [87] were used to remove PETs of self-ligation, re-ligation, and dangling. The iterative correction matrixes of different resolutions were generated using HiC-Pro [88], and the TADs and TAD boundaries at 5-kb resolution were identified using HiCExplorer [75]. The TAD boundaries located within 10 kb were considered conserved between ear and tassel. Loop calling was performed using Juicer HICCUPS [89] with 5-kb and 10-kb bin size and default parameters. The aggregate enrichment of peaks was measured by Juicer Aggregate Peak Analysis (APA) [89] at 5-kb resolution. Chromatin interaction heatmaps were produced using Juicebox [90].

### ATAC-seq library preparation

The ATAC-seq libraries were constructed according to the previous study with some modifications [91]. Intact nuclei were isolated from ~ 10 fresh ears and tassels as described in Hi-C library preparation. After filtration through Miracloth, Triton X-100 was added to a final concentration of 0.3% and the samples were incubated for 10 min on ice. The nuclei were pelleted by centrifugation and were resuspended in 20 μl 1× TTBL buffer (VAHTS, TD501), and the integrity and amount of nuclei were controlled before processing. For each library, ~ 10,000 nuclei were treated with Tn5 (VAHTS, TD501) in the presence of 0.3% Triton X-100. The transposition reaction was carried out at 37 °C for 30 min. Then, the sample was immediately purified using a Qiagen MinElute kit and the purified fragments were amplified for 10–13 cycles to construct a library, according to the instructions (VAHTS, TD501). The amplicons were subjected for size selection using DNA clean beads (KAPA) and for paired-end sequencing (2 × 150 bp) using Illumina Hiseq X-Ten.

### RNA-seq, BS-seq, and ChIP-seq library preparation

Total RNA of ears and tassels were extracted using Direct-zol RNA Miniprep (Zymo Research), and libraries were constructed using NEBNext Ultra Directional RNA Library Prep Kit according to the instructions; BS-seq libraries were constructed as previously described [92]. About 50 (for each replicate) cross-linked ears or tassels were used to construct ChIP-seq libraries as previously described [10].

### RNA-seq, BS-seq, ATAC-seq, and ChIP-seq data analysis

The quality of raw sequencing data was controlled using Trimmomatic with parameters “LEADING:20 TRAILING:20 SLIDINGWINDOW:4:15 MINLEN:20” [93]. The clean data of RNA-seq was mapped to the genome using HISAT2 [94] with the default settings. The uniquely mapped reads (mapping quality > 10) were used to calculate the gene expression levels, and differentially expressed genes were called using Cufflinks programs [95]. BS-seq data was processed using Bismark [96], and the ATAC-seq and ChIP-seq clean data was mapped to the genome using bowtie2 [97] with the default parameters. After removing PCR duplicates and low-quality reads (mapping quality score < 10) using samtools [98], we used MACS2 [99] to call peaks with parameters (ATAC-seq: --shift -100 --extsize 200 --nomodel -B -SPMR; H3K4me3: default parameters; H3K9ac and H3K27me3: --broad --broad-cutoff 0.05).

### GO analysis

GO analysis was performed using Agrigo v2.0 [56] (http://systemsbiology.cau.edu.cn/agriGO v2/index.php).

### Analysis of dynamic chromatin states

We used DiffBind [61] to call the differential peaks of ATAC-seq, H3K4me3, and H3K27me3 between ear and tassel with default parameters. These differential peaks included local and distal peaks and were regarded as differential enrichment of OCRs, H3K4me3, and H3K27me3 modifications. Then, we divided these differential enriched features into ear- and tassel-enriched OCRs and H3K4me3, and ear- and tassel-depleted H3K27me3. The BEDtools were applied [100] to identify adjacent genes of the differential peaks, which were located within 3 kb upstream of transcription start sites and the transcription termination sites of genes. The Venn diagrams were plotted in http://bioinformatics.psb.ugent.be/webtools/Venn/. We used chromHMM [101] for chromatin state clustering. All genome browsers were plotted using pyGenomeTracks [75].

### TF ChIP-seq and DAP-seq analysis

ChIP-seq data for TB1, FEA4, and KN1 were downloaded from NCBI SRA or GEO (accession numbers PRJNA517683, GSE61954, and GSE39161). Reads were mapped to the maize genome version 4 using bowtie2 [97]. After removing duplicates, the bam files were used to generate the normalized (RPKM) bigwig files for genome browser plots. MACS2 was used to call peaks for TB1 with default parameters, and only peaks present in both replicates were used for analysis. For FEA4, KN1, and ARFs (DAP-seq GEO accession: GSE111857), the processed peak files were obtained from the GEO accessions, respectively, and the peak coordinates were transferred to maize genome version 4 using assembly converter in the EnsemblPlants database. Only peaks present in both replicates for FEA4 and in both ear and tassel data for KN1 were used for analysis. The ChIP-seq data generated from leaf protoplast was obtained from the pre-release maize cistrome data collection (http://www.epigenome.cuhk. edu.hk/C3C4.html), and the TF browser tracks were obtained from JBbrowse at the website above.

### Motif analysis of OCRs and TAD boundaries

For LoOCRs and dOCRs, they were adjusted to the same size (200 bp) centered peak summits and de novo motif discoveries of these loci sequences were performed using DREME in the MEME suite with default settings. We selected the fully conserved TAD boundaries between ear and tassel for accuracy and focused on the motifs associated with OCRs in conserved TAD boundaries, given that the interactions between potential structural proteins and DNA are more likely located in easily accessible chromatin regions [23]. These OCRs were then divided into two categories as previously described [75]: promoter OCRs (OCRs overlapping with regions between 1 kb upstream and 200 bp downstream of transcription start sites) and the remaining non-promoter OCRs. We randomly selected the same number of promoter OCRs and non-promoter OCRs outside of TAD boundaries as background. All the OCRs were adjusted to the same size (200 bp) centered peak summit. The 4 sequence datasets were subjected to the de novo motif analysis separately using MEME-chip with default settings [102].

### Co-expression analysis of gene pairs with chromatin loops

To investigate the correlation for gene pairs in chromatin loops, the Pearson correlation coefficient (PCC) of gene pairs was calculated using RNA-seq datasets of 65 different tissues from B73 [103]. As a control, we randomly selected gene pairs with similar chromatin distances and calculated the PCCs. The real gene pairs and randomly selected gene pairs were then assigned to 1000 batches, and the average PCCs of each batch were used for box plots.

## Supplementary information

**Additional file 1: Figure S1.** Reproducibility analysis between replicates. **Figure S2.** Mapping of OCRs and epigenome marks. **Figure S3.** GO analysis of DEGs and dynamic LoOCRs and chromatin modifications associated with differential gene expression between ear and tassel. **Figure S4.** OCRs can be a platform for TF binding. **Figure S5.** Quality control of Hi-C data. **Figure S6.** Characterization of TADs. **Figure S7.** De novo motif analysis of TAD boundaries. **Figure S8.** Characterization of chromatin loops. **Figure S9.** Interaction between genes and their genetically mapped regions. **Figure S10.** Characterization of dOCR-gene loops in which some known genes are involved. **Figure S11.** Dynamic activities of dOCRs contribute to tissue-specific gene expression. **Figure S12.** Epigenetic features around distal trait-associated SNPs.

**Additional file 2: Table S1.** Summary of data generated in this study. **Table S2.** Open chromatin regions in ear. **Table S3.** Open chromatin regions in tassel. **Table S4.** Differentially expressed gene with their changed epigenetic feature and bound TF in ear and tassel. **Table S5.** Differential OCRs. **Table S6.** Differential H3K4me3. **Table S7.** Differential H3K27me3. **Table S8.** Genes co-bound by TFs in ear. **Table S9.** Chromatin loops in ear. **Table S10.** Chromatin loops in tassel. **Table S11.** dOCR-gene loops in ear. **Table S12.** dOCR-gene loops in tassel. **Table S13.** Ear-active dOCR-gene loops. **Table S14.** Tassel-active dOCR-gene loops. **Table S15.** The number of genes looped by dOCRs. **Table S16.** The number of tissue-enriched genes looped by tissue-active dOCRs. **Table S17.** Significant ear and tassel trait-associated SNPs used for analysis.

**Additional file 3.** Review history.

## Data Availability

All the sequencing data generated in this study have been deposited in NCBI SRA under the accession number PRJNA599454 [104]. The ChIP-seq data for FEA4 (GSE61954), KN1 (GSE39161), and TB1 (PRJNA517683) were downloaded from NCBI GEO or SRA [64–66]. The ChIP-seq data generated from leaf protoplast was obtained from the pre-release maize cistrome data collection (http://www.epigenome.cuhk. edu.hk/C3C4.html). The ear and tassel trait-associated intergenic SNPs were obtained from a recent review [76].
