# Supplementary material for: 3D genome architecture coordinates trans and cis regulation of differentially expressed ear and tassel genes in maize
Source: Genome Biol. 2020 Jun 16;21:143. doi: 10.1186/s13059-020-02063-7 (PMC7296987; doi:10.1186/s13059-020-02063-7)
Supplement: Supplementary file 1 — Additional file 1: Figure S1. Reproducibility analysis between replicates. Figure S2. Mapping of OCRs and epigenome marks. Figure S3. GO analysis of DEGs and dynamic LoOCRs and chromatin modifications associated with differential gene expression between ear and tassel. Figure S4. OCRs can be a platform for TF binding. Figure S5. Quality control of Hi-C data. Figure S6. Characterization of TADs. Figure S7. De novo motif analysis of TAD boundaries. Figure S8. Characterization of chromatin loops. Figure S9. Interaction between genes and their genetically mapped regions. Figure S10. Characterization of dOCR-gene loops in which some known genes are involved. Figure S11. Dynamic activities of dOCRs contribute to tissue-specific gene expression. Figure S12. Epigenetic features around distal trait-associated SNPs. [file 13059_2020_2063_MOESM1_ESM.pdf]

3D genome architecture coordinates trans and cis regulation of differentially expressed ear and tassel genes in maize

Yonghao Sun[1†], Liang Dong[1†], Ying Zhang[1], Da Lin[2], Weize Xu[2], Changxiong Ke[1], Linqian Han[1], Lulu Deng[2], Guoliang Li[1], David Jackson[1,3], Xingwang Li[1]*, Fang Yang[1]*

[1]National Key Laboratory of Crop Genetic Improvement, Huazhong Agricultural University, Wuhan 430070, PR China.

[2]State Key Laboratory of Agricultural Microbiology, Huazhong Agricultural University, Wuhan, China.

[3]Cold Spring Harbor Laboratory, Cold Spring Harbor, New York 11724, USA.

* Correspondence: xingwangli@mail.hzau.edu.cn; fyang@mail.hzau.edu.cn

†Yonghao Sun and Liang Dong contributed equally to this work.

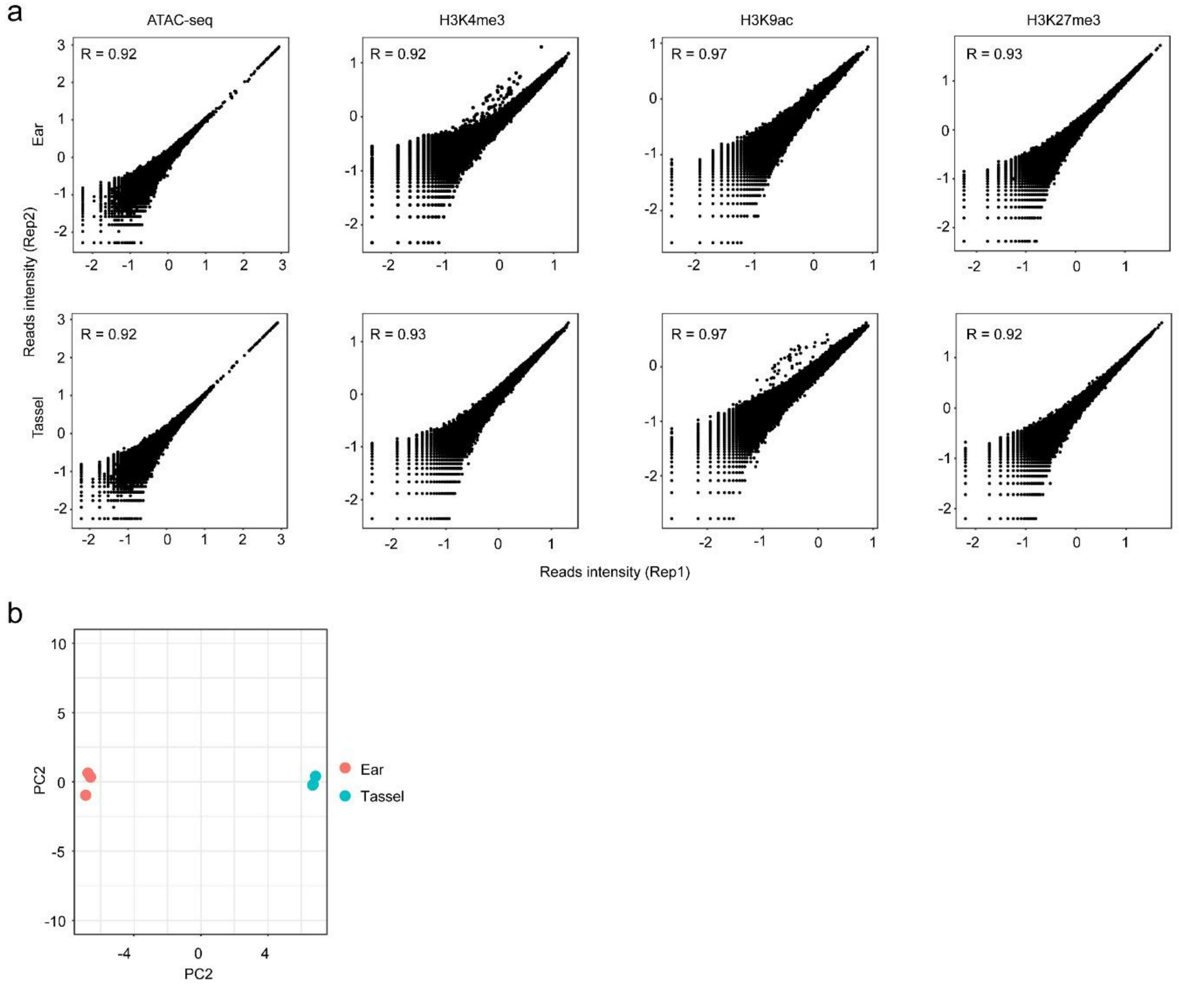

Fig. S1. Reproducibility analysis between replicates. a Correlation analysis of reads intensity between replicates for ATAC-seq and histone ChIP-seq (H3K3me3, H3K9ac and H3K27me3). The reads number per 10kb was used for read intensity evaluation. *Pearson's correlation coefficients* (R) are shown at the upper left corner. **b** PCA analysis of RNA-seq data of ear and tassel. Each tissue contains 3 biological replicates.

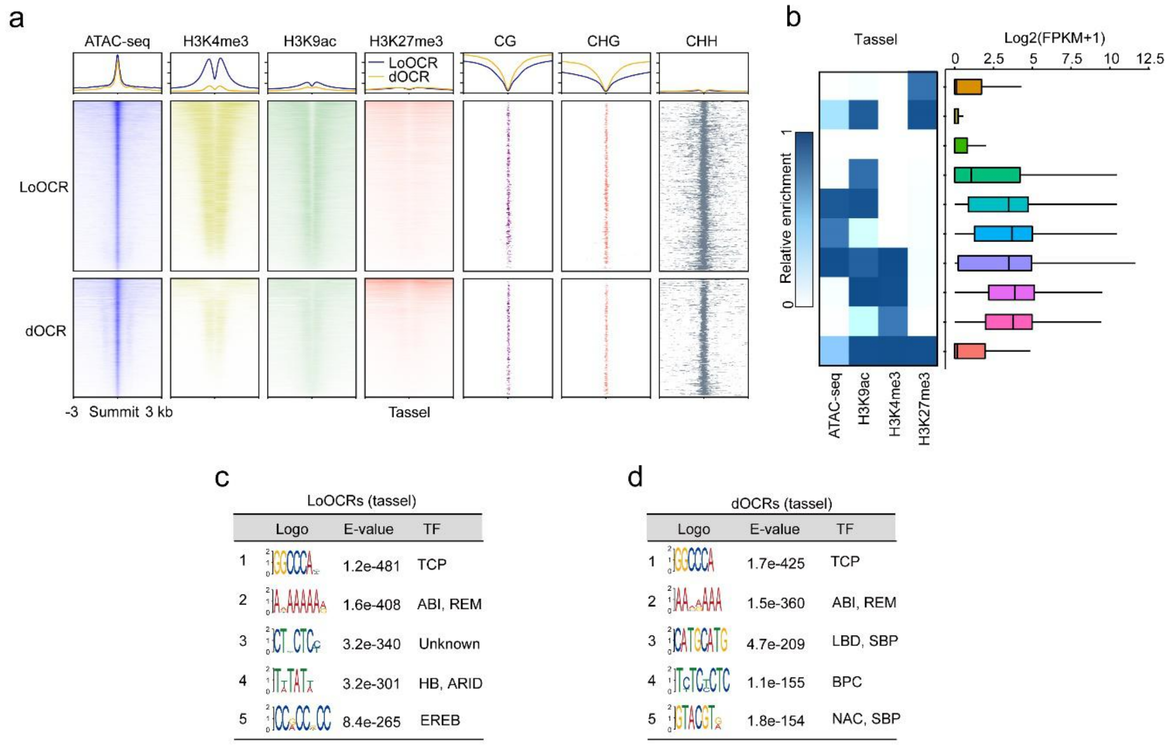

Fig. S2. Mapping of OCRs and epigenome marks. a Epigenome profiles at LoOCRs and dOCRs centred on OCR summits in tassel. Shown are ± 3 kb from ATAC-seq peak summits. **b** Gene expression levels correspond to different clusters of chromatin accessibility and histone modifications in tassel. **c, d** DNA motifs enriched in LoOCRs (**i**) and dOCRs (**j**) of tassel. The corresponding candidate motif-binding TFs are shown.

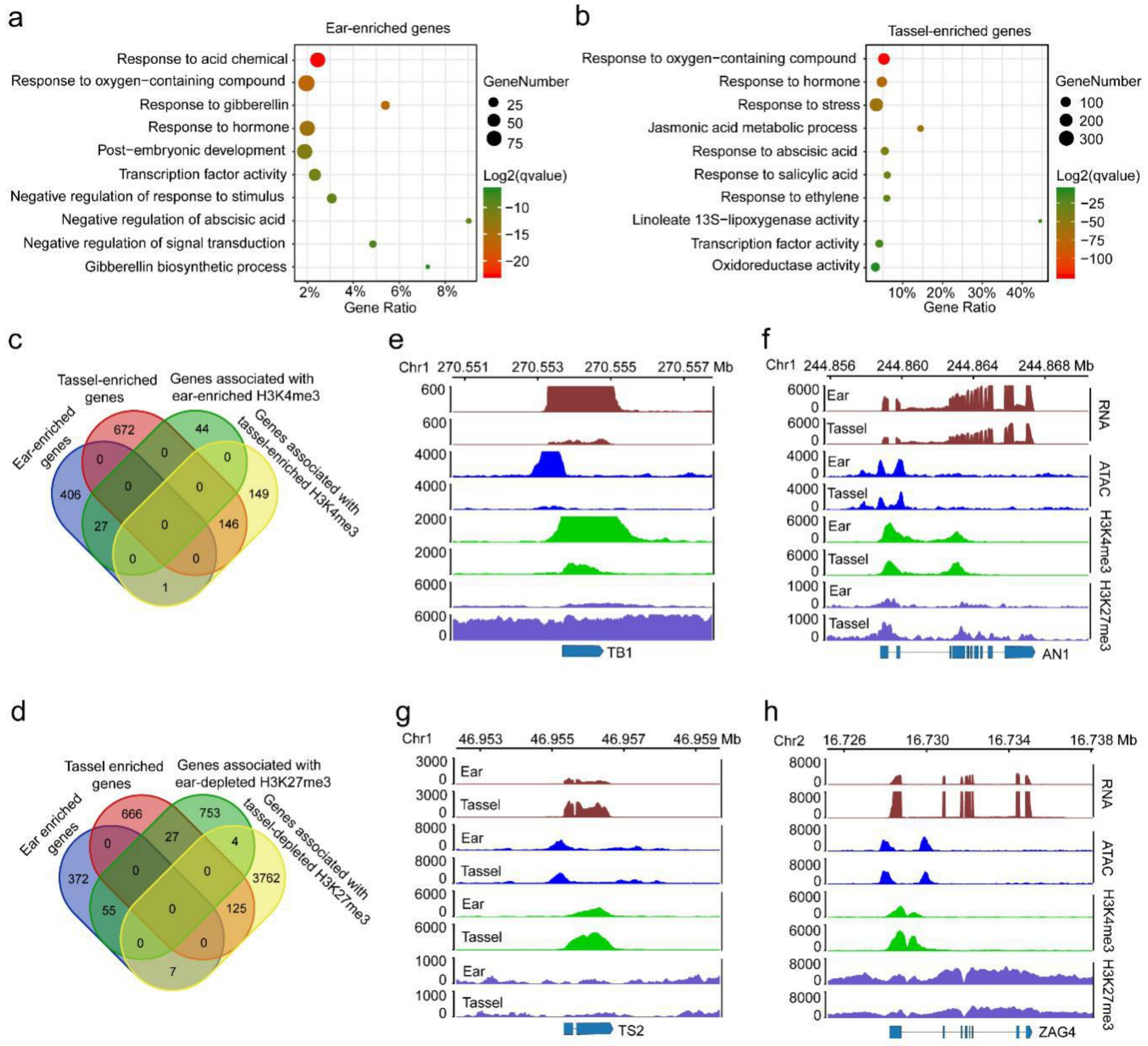

Fig. S3. GO analysis of DEGs and dynamic LoOCRs and chromatin modifications associated with differential gene expression between ear and tassel. a, b GO analysis of ear-enriched genes (a) and tassel-enriched genes (**b**). **c, d** Venn diagram of overlaps between tissue-enriched gene sets and genes associated with tissue-enriched H3K4me3 (**c**) and tissue-depleted H3K27me3 (**d**). **e-h** Examples of ear-enriched genes *TB1* (**e**) and *AN1* (**f**), and tassel-enriched genes *TS2* (**g**) and *ZAG4* (**h**) with corresponding chromatin feature changes between ear and tassel.

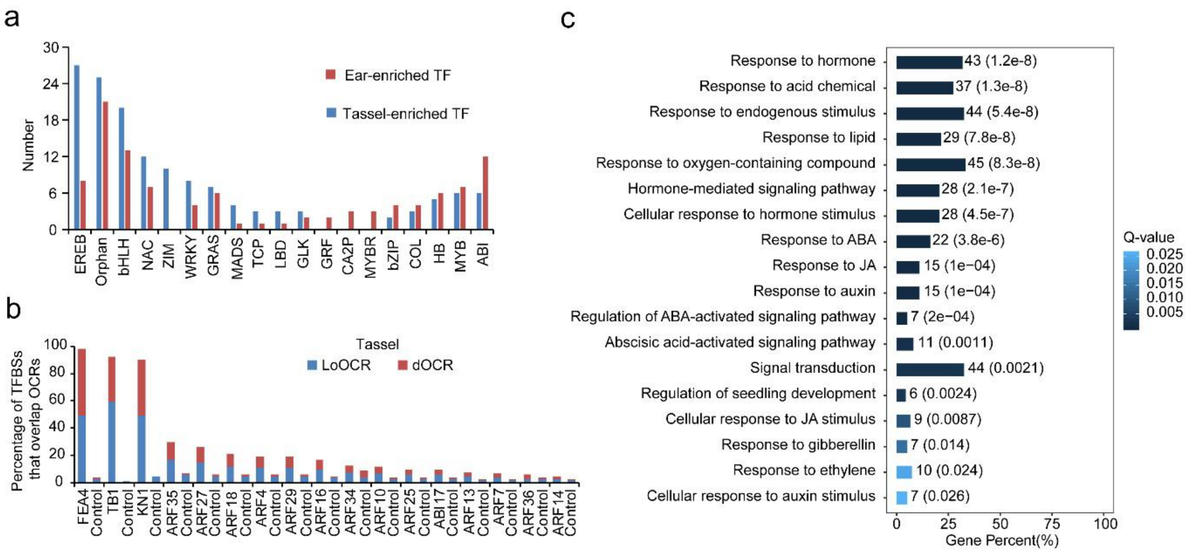

Fig. S4. OCRs can be a platform for TF binding. a The number of differentially expressed TFs belonging to different families in ear and tassel. **b** The percentages of 3 TFs binding sites from in vivo ChIP-seq and 14 TFs binding sites from in vitro DAP-seq locating in LoOCRs and dOCRs in tassel. Controls: the same number of local and distal regions as LoOCRs and dOCRs, respectively, by random shifting. **c** GO analysis of *TB1* targeted ear-enriched genes identified by ChIP-seq (only genes with TB1 binding sites in their LoOCRs were considered).

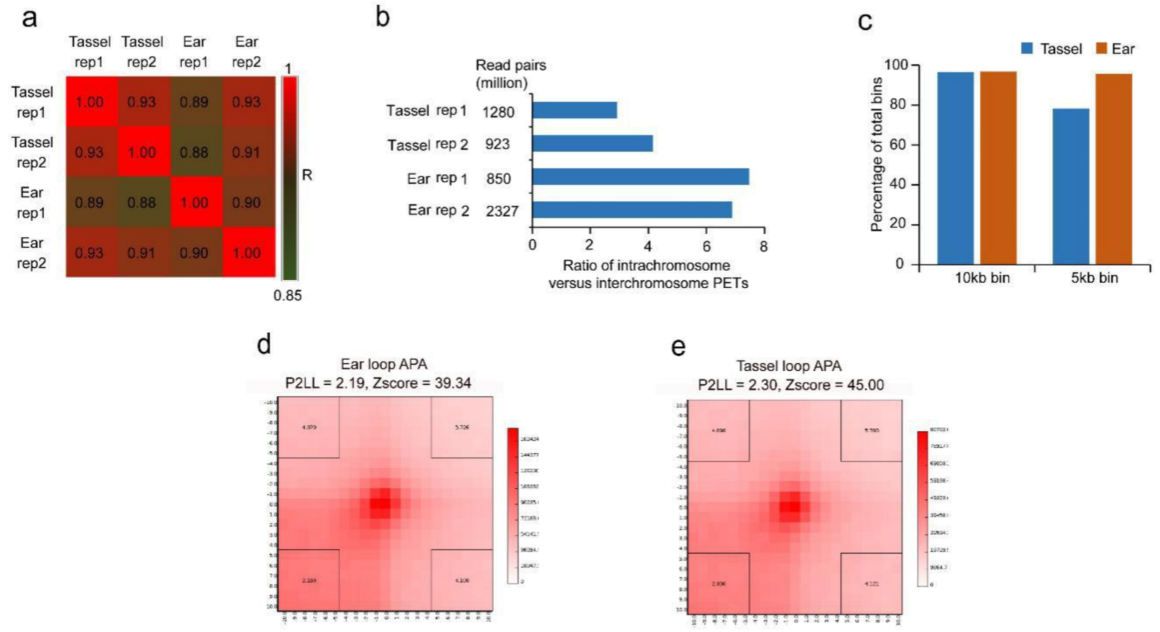

Fig. S5. Quality control of Hi-C data. a The correlations between replicates in ear and tassel, respectively, were calculated by HiCRep at 80 kb resolution for ear and tassel. **b** The ratio of intrachromosome versus interchromosome PETs in our Hi-C data. The raw read pair numbers of each replicate are shown in the middle. **c** To assess the resolution of our Hi-C data, the PET numbers for each bin at 10-kb and 5-kb resolution were calculated as described in Rao et al. (2014), which requires that the top 80% of bins should have no less than 1,000 contacts. Although 78.3% of bins in tassel have more than 1000 contacts, we considered the maps of ear and tassel as 5-kb resolution. **d, e** Standard APAs (Aggregate Peak Analysis) of chromatin loops (5kb resolution) of ear (**d**) and tassel (**e**).

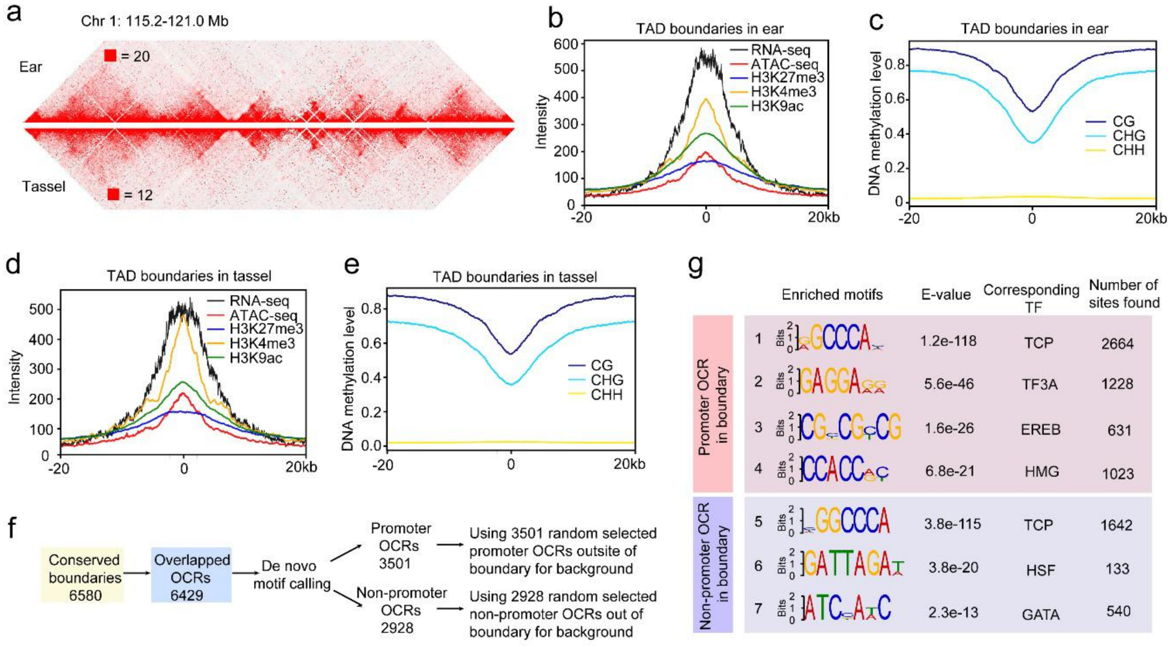

Fig. S6. Characterization of TADs. a Clear TADs were detected in ear and tassel on a region of chromosome 1 from our chromatin interaction map. **b-e** Average enrichments of different chromatin features around TAD boundaries of ear and tassel. RNA profiles, ATAC states and histone modifications are shown in (**b, d**), and CG, CHG and CHH methylation features are shown in (**c, e**). **f** Pipeline of the strategy used to identify de novo motifs at TAD boundaries. **g** Several TF binding DNA motifs enriched at the promoter and non-promoter TAD boundaries. The E-value, corresponding motif binding TFs and motif numbers are shown.

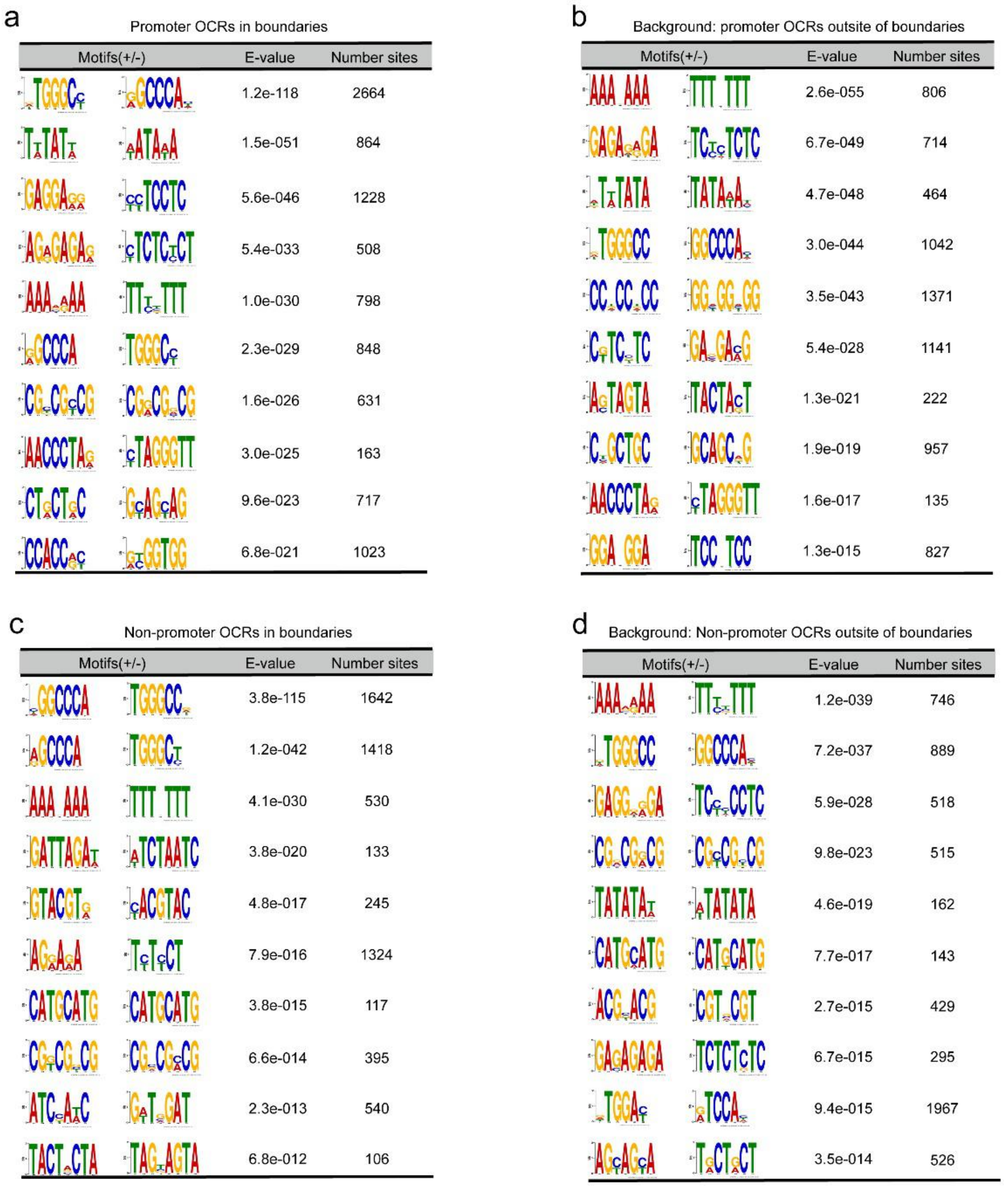

Fig. S7. De novo motif analysis of TAD boundaries. The enriched DNA motifs associated with promoter OCRs (**a**) and non-promoter OCRs (**c**) in TAD boundaries along with their corresponding backgrounds (promoter OCRs (**b**) and non-promoter OCRs (**d**) outside of TAD boundaries). Only top 10 of the enriched DNA motifs are shown.

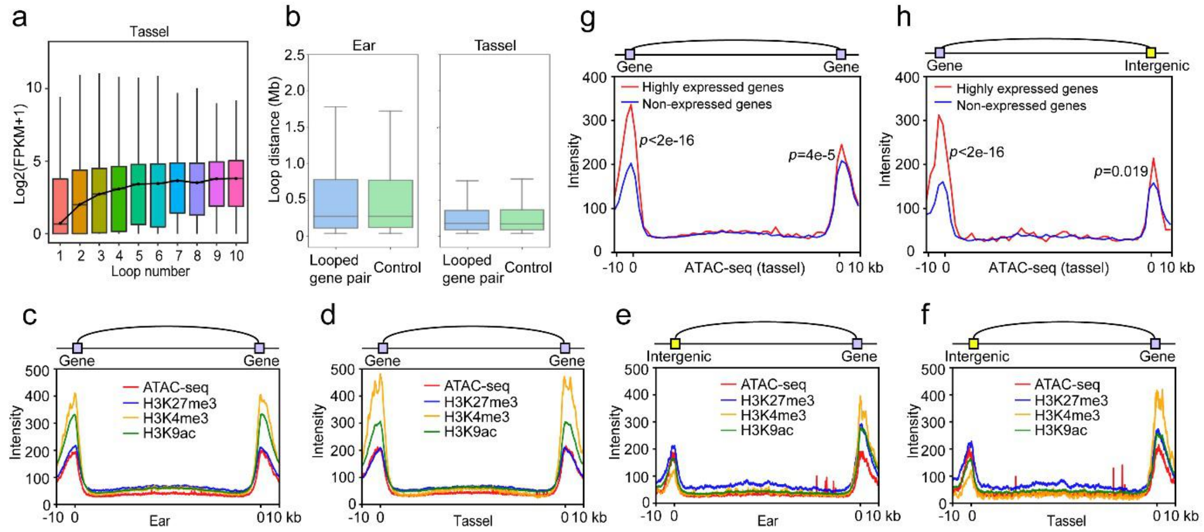

Fig. S8. Characterization of chromatin loops. a The correlation between gene expression level and the number of loops in tassel. **b** The distance of randomly selected gene pairs (control) used for co-expression analysis is similar to that of gene pairs with real chromatin loops. **c-f** Average distributions of different epigenetic features for gene-gene loops in ear (**c**) and tassel (**d**) and intergenic-gene loops in ear (**e**) and tassel (**f**), respectively. **g, h** The average epigenetic features of gene-gene loops showing that highly expressed genes have higher chromatin accessibility together with their looped regions than non-expressed genes in tassel (**g**). The intergenic-gene loops have a similar tendency (**h**). Highly expressed genes: FPKM > 100; non-expressed genes: FPKM < 1. The target genes and their looped regions are on the left and right sides of the loops, respectively, in the diagram. Only loops with a distance < 500 kb are included. The Wilcoxon test was used to test significance.

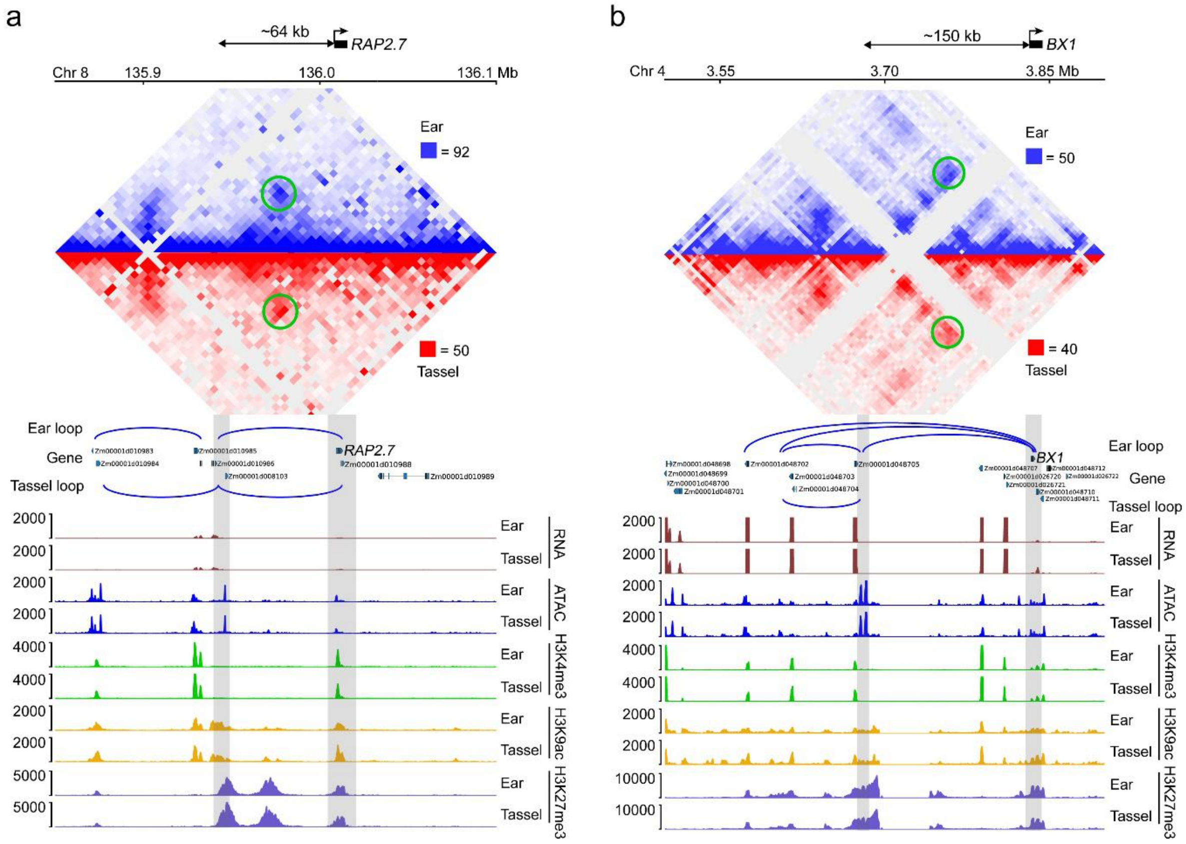

Fig. S9. Interaction between genes and their genetically mapped regions. Hi-C loops detect interactions between genes *RAP2.7* (**a**), *BX1* (**b**) and their genetically mapped regions. Grey shadings mark the interaction regions and green circles mark the loops in the heatmaps.

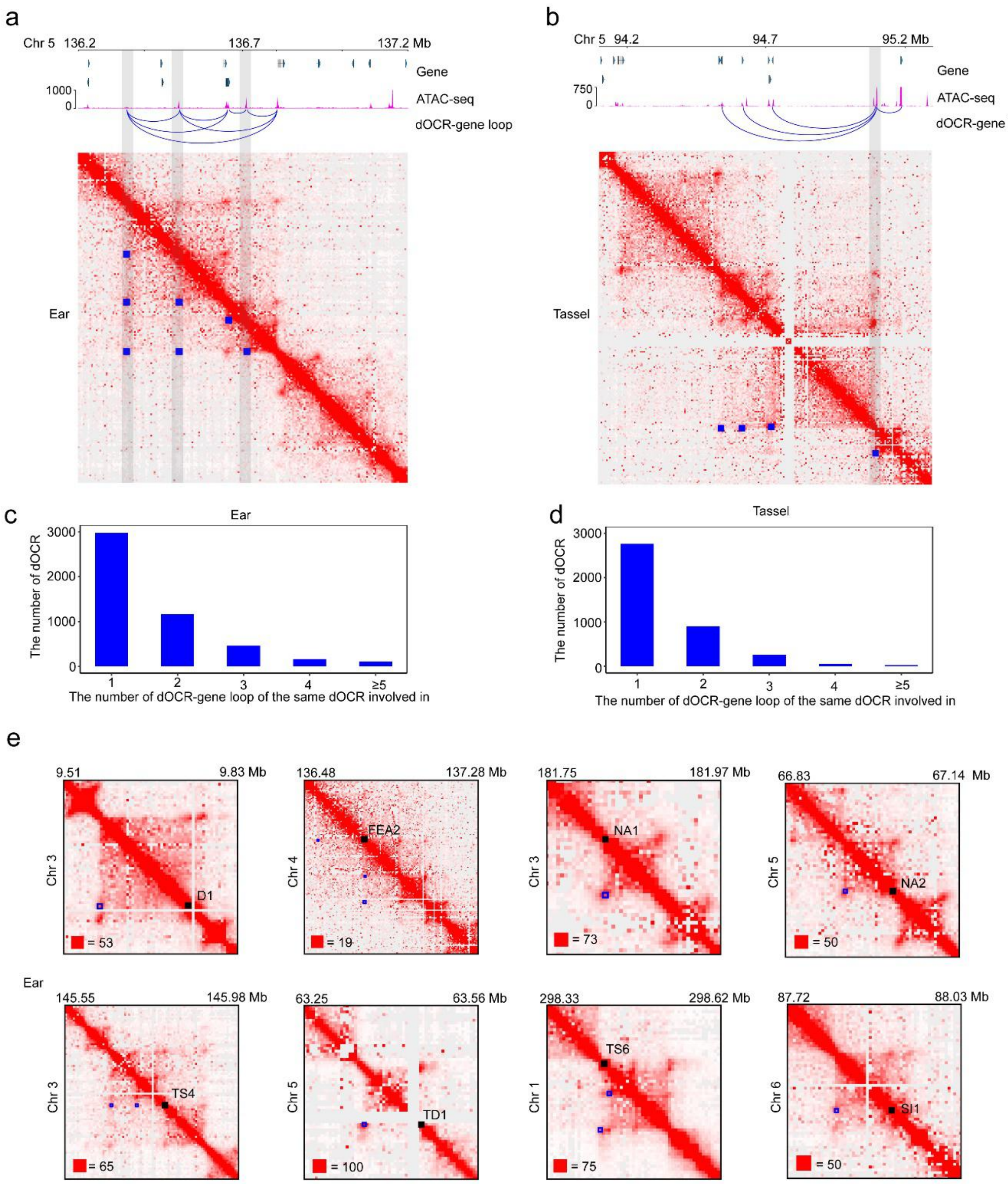

Fig. S10. Characterization of dOCR-gene loops in which some known genes are involved. a,b Examples of one dOCRs looping with multiple genes in ear (**a**) and tassel (**b**). Grey shadings mark the regions of dOCRs looping with multiple genes. Blue boxes mark the loops on the chromatin interaction maps. **c, d** The distribution of dOCRs (y-axis) involved in various number of dOCR-gene loops (x-axis) in ear (**c**) and tassel (**d**). **e** Examples of key inflorescence regulation genes involved in chromatin loops (blue boxes) in ear. Gene loci are marked by black dots.

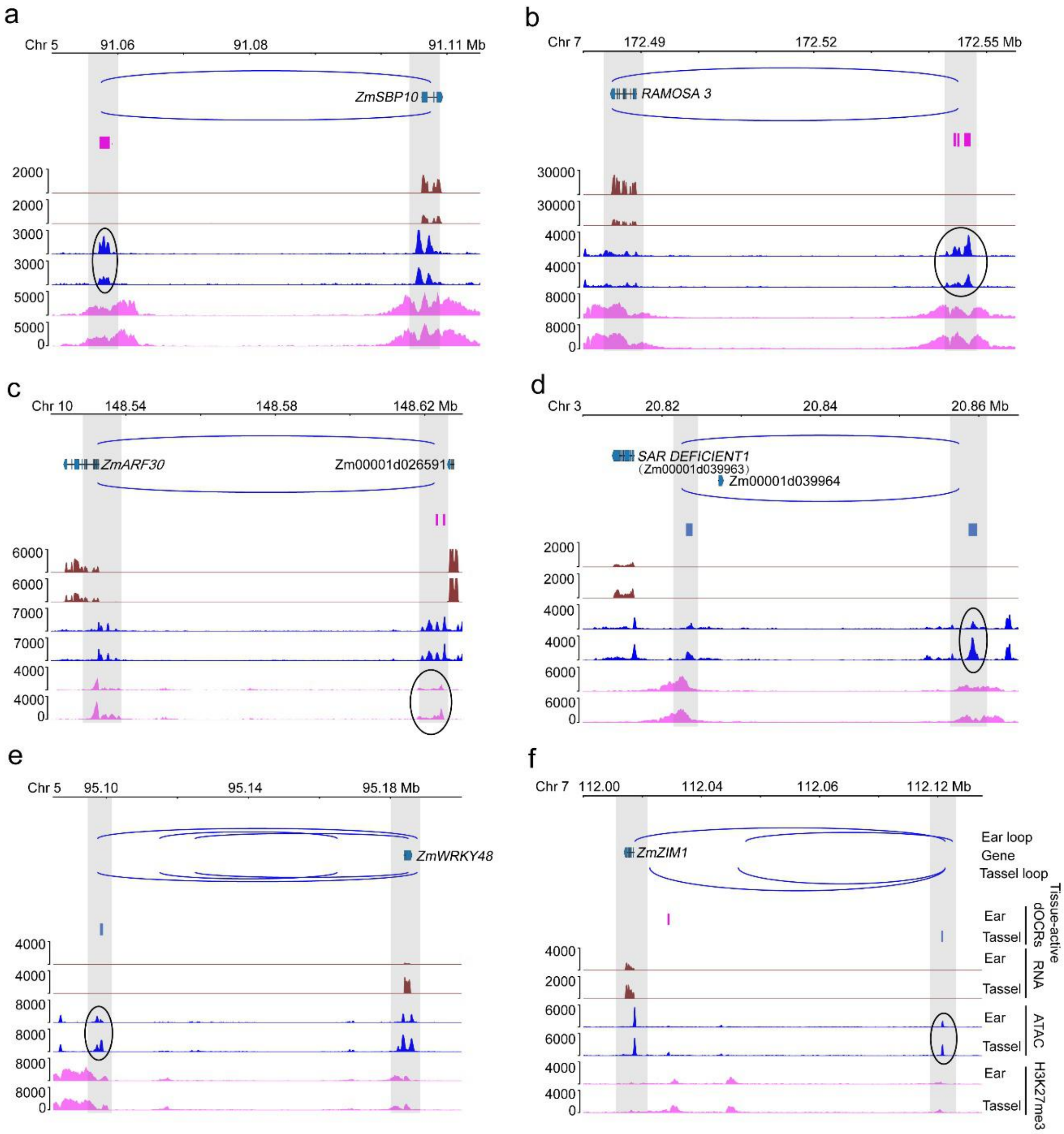

Fig. S11. Dynamic activities of dOCRs contribute to tissue-specific gene expression. a-c Examples of ear-active dOCRs looping 3 TFs, including *ZmSBP10* (**a**), *RA3* (**b**) and *ZmARF30* (**c**), which have higher expression in ear than in tassel. Black circles mark the dOCRs with variant activities. **d-f** Examples of tassel-active dOCRs looping 3 JA-related genes, including *SAR DEFICIENT1* (**d**), *ZmWRKY48* (**e**) and *ZmZIM1* (**f**), which have a higher expression in tassel than in ear. Black circles mark the dOCRs with variant activities.

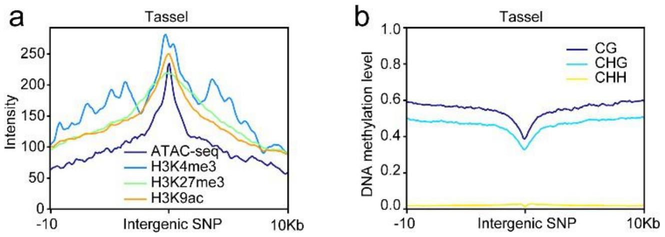

Fig. S12. Epigenetic features around distal trait-associated SNPs. The average distribution of ATAC, histone modifications (**a**) and DNA methylation (CG, CHG, CHH) level (**b**) centred on the trait-associated distal SNPs in tassel. 41,710 distal SNPs were used for calculation.
