# Supplementary material for: 3D genome architecture coordinates trans and cis regulation of differentially expressed ear and tassel genes in maize
Source: Genome Biol. 2020 Jun 16;21:143. doi: 10.1186/s13059-020-02063-7 (PMC7296987; doi:10.1186/s13059-020-02063-7)
Supplement: Supplementary file 3 — Additional file 3. Review history. [file 13059_2020_2063_MOESM3_ESM.docx]

Review History

**First round of review**

**Reviewer 1**

**Are you able to assess all statistics in the manuscript, including the appropriateness of statistical tests used?**

Yes, and I have assessed the statistics in my report.

**Comments to author:**

In this manuscript, the authors compared the structural and epigenetic features of chromatin in the developing maize ear and tassel tissues. To this end, they generated and compared ATAC-seq, BS-seq, Hi-C, and ChIP-seq (H3K4me3, H3K9ac, and H3K27me3) datasets in these two tissues. They also associated their OCR annotation (derived from ATAC-seq) with other TF ChIP-seq data. Together, this manuscript provides meaningful insights into the mechanisms underlying differential gene expression in distinct plant tissues. My comments are listed as follows, among which the one marked with ** is my major concern.

Line #100, "importantly,…": this statement needs a reference.

Fig 1C, the annotation of an OCR is either "local (within 3kb upstream of TSS, LoOCR)" or "distal (dOCR)". How about OCRs residing inside gene body, and OCRs located at 3' downstream regions of genes?

Fig1f lacks a color key. Nonetheless, I assume that dark blue means "high" and white/light blue mean "low". It is rather interesting that the gene group with the highest expression is the one with a high level of H3K27me3 and an intermediate level of ATAC-seq signal (the bottom row). This pattern is true for both the ear and tassel tissue, any explanation?

Please be more precise about Fig 1i and 1j, were these motifs identified from the combined OCR datasets of ear and tassel? (or their shared OCRs)

Fig 1i, BPC TFs bind GA (or TC) repeats; however, the third one does not seem to show this pattern.

Regarding the part in lines #186-196, let us consider it from the opposite direction: what is the fraction of genes that do not show differential gene expression but has differential histone marks or chromatin accessibilities? Is it true that differential epigenetic marks elicit differential gene expression?

** lines 204-205: please note that not all these data were made from ear/tassel. The FEA4 ChIP-seq study from Pautler et al. only reported ear tissues; while the TB1 ChIP-seq data was based on vegetative tiller buds. Caution should be paid when one correlates ChIP-seq data from one tissue to other chromatin data (e.g., ATAC-seq) generated from another tissue. In this case, although I feel that the findings presented in Fig3, which are primarily based on such association analyses, are correct, I am not convinced. What if the differences in TF's chromatin binding patterns among different tissues were under-estimated? Note that the KN1 ChIP-seq peaks identified in ear and tassel had a huge difference (demonstrated in Fig S1 of Bolduc et al. 2012). This comment also applies to line#225. The cistrome database was based on transient TFs expression in protoplast (https://www.biorxiv.org/content/10.1101/2020.01.07.898056v1). It is misleading to display these TF ChIP-seq tracks with other histone features in Fig5, and the proposed model this figure (Fig5g) remains questionable.

Line#290, according to FigS 8j and 8h, the differences are moderate, please revise.

For examples shown in Fig4o, what tissue do they belong to? Do these selected genomic regions show different Hi-C maps in ear and tassel?

Line #333, "a genetically mapped distal region…": is this region overlapping the one highlighted in Fig 5f?

Line #344-346, this sentence is related to the binding of multiple TFs to dOCRs, please see my major comment.

Are there any connections between DMRs (differentially methylated regions) and differentially accessible chromatin regions or DEGs?

**Reviewer 2**

**Are you able to assess all statistics in the manuscript, including the appropriateness of statistical tests used?**

Yes, and I have assessed the statistics in my report.

**Comments to author:**

This paper provides a high quality, rich resource that will be a key enabling resource for the maize genetics community.  It provides a significant advance over previous studies and will be used by others to further examine the complex regulation of genes that specify the ear and tassel in maize. It also nicely ties to previous results on specific transcription factors that function in the ear and tassel. The paper is of broad interest to the biology community and especially the maize community.

Comments:

1.  The methods are appropriate, although, other than the ATAC-seq data, I am not sure if all of the datesets were replicated? This should be clearly stated in the materials and methods. and/or the results section.

2.  It seems like a single cell-based approach would have resulted in an even more robust dataset and would be more specific with regards to the various OCR, chromatin marks, etc. and their impact on gene regulation in both the ear and tassel.

3. On page 6 line 146 it says "significantly more active than those with repressive H3K27me3 or lacking modifications". One of the genes associated with H3K27me3 also had other marks (H3K4me3, H3K9me3) and exhibited high expression. I think this should be clarified.

4. There are several places in the text where it is stated that the comparison was "significant" or something like "exhibited more active chromatin and active H3K4me3 ... " (page 7 line 174-175) and gene expression differences (page 7 178-179). A statistical test should be shown in these cases.  

5. On page 11-12 Lines 283-284 a two-tailed test is performed on randomly selected gene pairs, I think an FDR should be shown here.

6. In figure 2 and in the corresponding results section, why wasn't the H3K9me3 data described?

7. In the gene co-expression network (Figure 3h) the authors used 65 RNA-seq datasets from B73. Since these datasets are not from just the ear and tassel, it may be a stretch to say they interact in the tassel. In addition, in the figure 3 legend it says that these are potentially regulated genes. I am unclear if these genes were shown to regulate it other. Without further data the only that can be said is that the genes are co-expressed.

**Authors Response**

**Point-by-point responses to the reviewers’ comments:**

*We would like to thank both reviewers for their excellent comments. We have fully addressed all the
concerns and comments raised by both reviewers. These comments are of help to significantly improve the manuscript. We highlighted in blue all the sentences revised in the new version of the manuscript.*

Reviewer #1: In this manuscript, the authors compared the structural and epigenetic features of chromatin in the developing maize ear and tassel tissues. To this end, they generated and compared ATAC-seq, BS-seq, Hi-C, and ChIP-seq (H3K4me3, H3K9ac, and H3K27me3) datasets in these two tissues. They also associated their OCR annotation (derived from ATAC-seq) with other TF ChIP-seq data. Together, this manuscript provides meaningful insights into the mechanisms underlying differential gene expression in distinct plant tissues. My comments are listed as follows, among which the one marked with ** is my major concern.

Comment 1.1 Line #100, "importantly,…": this statement needs a reference.

*Response 1.1 We have added the relevant references in the revised manuscript. Meanwhile, we have revised this sentence to “Therefore, the temporally and spatially specific expression of related genes contributes to the morphological difference and sex determination in ear and tassel [29, 31, 32, 35, 38, 40]” (see lines 101-102).*

Comment 1.2 Fig 1C, the annotation of an OCR is either "local (within 3kb upstream of TSS, LoOCR)" or "distal (dOCR)". How about OCRs residing inside gene body, and OCRs located at 3' downstream regions of genes?

*Response 1.2 The local OCRs (LoOCRs) were defined as the open chromatin regions from 3 kb upstream of transcription start sites (TSS), spanning the gene body, to transcription termination sites (TTS) of genes, so the OCRs residing inside the gene body belong to LoOCRs. The OCRs located at 3' downstream regions of genes were defined as distal OCRs (dOCRs). We defined LoOCRs and dOCRs based on the previous studies on DNase I hypersensitive sites (DHSs) identification in Arabidopsis (Zhang et al., 2015; Yan et al., 2019). We apologize for the confusion raised due to unclear definition of LoOCRs in main text. We have corrected this in the revised manuscript (refer to lines 129-130).*

Comment 1.3 Fig1f lacks a color key. Nonetheless, I assume that dark blue means "high" and white/light blue mean "low". It is rather interesting that the gene group with the highest expression is the one with a high level of H3K27me3 and an intermediate level of ATAC-seq signal (the bottom row). This pattern is true for both the ear and tassel tissue, any explanation?

*Response 1.3 Thank you for pointing out this. We have added the color key in Fig 1f and Figure S2b. Yes, you are right that dark blue means "high" and white/light blue means "low". The Reviewer raises an interesting question. We reanalyzed the data and found that there was a mistake during the figure plot on the right panel due to an error of data sorting. The expression values were previously plotted as 10, 1, 2, 3…9, whereas the correct order should be 1, 2, 3…10. Therefore, the gene cluster with high H3K27me3 (the bottom row) has in fact a low expression level. Meanwhile, our other conclusions are not affected. We apologize for this mistake and leading confusion. We have corrected this in the revised Figures (Fig 1f and Figure S2b).*

Comment 1.4 Please be more precise about Fig 1i and 1j, were these motifs identified from the combined OCR datasets of ear and tassel? (or their shared OCRs)

*Response 1.4 The motifs shown in Fig 1i and 1j are from ear LoOCRs and dOCRs, respectively. Based on your comments, we added the motifs enriched in tassel LoOCRs and dOCRs, respectively. We have added the tissue information in Fig 1i, j and the tassel motifs to Additional file 1 (Figure S2c, d) for clarity.*

Comment 1.5 Fig 1i, BPC TFs bind GA (or TC) repeats; however, the third one does not seem to show this pattern.

*Response 1.5 Thank you for pointing out this. We agree with the Reviewer that the third motif sequence in Fig 1i is different from BPC TFs binding repeats. We therefore changed the name of the third one from “BPC” to “unknown” in the panel.*

Comment 1.6 Regarding the part in lines #186-196, let us consider it from the opposite direction: what is the fraction of genes that do not show differential gene expression but has differential histone marks or chromatin accessibilities? Is it true that differential epigenetic marks elicit differential gene expression?

*Response 1.6 We appreciate your thought-provoking comments. We followed your idea and observed that ~50% of DEGs had at least one epigenetic feature change. We also revealed that there were a lot of genes with differential epigenetic features but had no differential expression. As we know, transcription regulation is a multiple-step process and depends on the control of many factors. Epigenetic features provided in our study are only part of the regulation process. In general, one or multiple epigenetic feature changes could induce the change of chromatin accessibility, which would affect the recruit of regulatory protein factors leading to gene expression change. Therefore, the epigenetic feature change not always leads to transcription change. In our results shown in Fig 2b-d, f, g, gene expression changes are correlated with the epigenetic feature changes. We also realized that using “elicit” might overstate the effects of epigenetic feature changes on differential gene expression, therefore replaced it with “are correlated with” at the relevant positions (lines 194-195, 198, 430) for more accuracy.*

Comment 1.7 ** lines 204-205: please note that not all these data were made from ear/tassel. The FEA4 ChIP-seq study from Pautler et al. only reported ear tissues; while the TB1 ChIP-seq data was based on vegetative tiller buds. Caution should be paid when one correlates ChIP-seq data from one tissue to other chromatin data (e.g., ATAC-seq) generated from another tissue. In this case, although I feel that the findings presented in Fig3, which are primarily based on such association analyses, are correct, I am not convinced. What if the differences in TF's chromatin binding patterns among different tissues were under-estimated? Note that the KN1 ChIP-seq peaks identified in ear and tassel had a huge difference (demonstrated in Fig S1 of Bolduc et al. 2012). This comment also applies to line#225. The cistrome database was based on transient TFs expression in protoplast (https://www.biorxiv.org/content/10.1101/2020.01.07.898056v1). It is misleading to display these TF ChIP-seq tracks with other histone features in Fig5, and the proposed model this figure (Fig5g) remains questionable.

*Response 1.7 We agree with the Reviewer that not all ChIP-seq data used in the paper were made from ear/tassel and the differences in TF's chromatin binding patterns among different tissues might be under-estimated. We used TB1 ChIP-seq data from vegetative tiller buds since we are not able to generate TB1 binding peaks from ear and tassel, although TB1 is expressed and has a similar growth-suppression function in both inflorescence and tiller. Based on the previous studies showing that open chromatin regions and in vitro DAP-seq data could be used to predict tissue-specific TF binding sites (O’Malley et al., 2017; Galli et al., 2018), we analyzed the correlation of binding peaks of FEA4 and KN1 captured in ear and tassel and TB1 binding peaks captured in tiller buds with our OCRs. We found more than 90% of the selected high confident TF binding peaks overlapping with OCRs both in ear and tassel (Fig. 3a, Figure S4b). In addition, > 65% of the 6 TFs binding sites generated from leaf protoplasts overlapped with tassel OCRs. We thus speculate that the overlapping sites might be the regulatory regions of these TFs present in ear and tassel.
We also agree with the Reviewer that the KN1 ChIP-seq peaks identified in ear and tassel have significant differences, we thus selected the common peaks of KN1 identified in both ear and tassel for overlapping analysis with OCRs. We didn’t perform differential analysis on their binding sites between ear and tassel. The differential expression of genes between ear and tassel were mainly explained by the differential expression of TFs themselves, which could affect their binding target genes expression, as shown in Figure 3h. Taken together, we speculate that binding sites of those TFs could be used for association analysis and the conclusion drawn should not be out of line. We also formulate the conclusion of this part in a speculative way (refer to lines 211-212 and 231-232) and discussed the limits in the Discussion section in the revised manuscript. Please refer to lines 440-443: “To be noted, using the ChIP-seq datasets generated from different tissues may under-estimate the differences of TF’s binding patterns in ear and tassel, although we only considered the high confident TF’s binding sites overlapping with ear/tassel OCRs for further analysis”.
We have clarified the tissue information in the revised manuscript (lines 433-435) and the relevant Figure panels (including Fig 3f, 3i, 5f, 5h) by labeling the tissues used for generating ChIP-seq data. In addition, we revised the proposed model in Fig 5g for more accuracy.*

Comment 1.8 Line#290, according to FigS 8j and 8h, the differences are moderate, please revise.

*Response 1.8 We agree with the Reviewer’s comment. We have revised the conclusion to “… showed moderately higher chromatin accessibility than that of non-expressed genes (p < 0.05, Wilcoxon test)” (lines 297-298) and added the significance test in Fig S8g, 8h and Fig 4i, 4j in the revised manuscript.*

Comment 1.9 For examples shown in Fig4o, what tissue do they belong to? Do these selected genomic regions show different Hi-C maps in ear and tassel?

*Response 1.9 The chromatin contact maps shown in Fig 4o are from ear. We have marked the tissue in figure and legend. We didn’t observe an obvious difference in the chromatin interaction maps of these selected genomic regions between ear and tassel.*

Comment 1.10 Line #333, "a genetically mapped distal region…": is this region overlapping the one highlighted in Fig 5f?

*Response 1.10 The genetically mapped distal region reported (Clark et al., 2006) is ∼58–69 kb upstream of the TSS of TB1. The highlighted region in Fig 5f harbors three dOCRs, which are located at ~59 kb, ~64 kb and ~68 kb upstream of the TSS of TB1 respectively. Therefore, these two regions are overlapping. We have clarified it in the main text (lines 344-346) and labeled this region in revised Fig 5f.*

Comment 1.11 Line #344-346, this sentence is related to the binding of multiple TFs to dOCRs, please see my major comment.

*Response 1.11 We agree with the Reviewer that we might under-estimate the difference because the tissues for ChIP-seq datasets of these TFs (leaf protoplasts) and our OCRs data (ear and tassel) are different. However, the binding sites of these TFs overlapped with the dOCRs of TB1, we here speculate that these TFs might bind to the OCRs of TB1 in ear or tassel. We rephrased the sentence the Reviewer mentioned to “Furthermore, we found that the binding sites of multiple TFs (TB1, FEA4, KN1, 3 ARFs, 2 bHLHs, 2 EREBs, 2 WRKYs, bZIP9 and HB79) overlapped with the dOCRs of TB1, suggesting that these TFs might bind to the distal cis elements of TB1 and form a regulatory module in ear and/or tassel" for more accuracy (lines 353-356). We also have labeled the tissues of these TFs in revised Fig 5f.*

Comment 1.12 Are there any connections between DMRs (differentially methylated regions) and differentially accessible chromatin regions or DEGs?

*Response 1.12 We thank the Reviewer for asking this and welcome the opportunity to clarify some results in DNA methylation data analysis. DMRs were identified as described previously (Garg et al., 2015). Then we investigated the relationship between differential chromatin accessibilities and DMRs in ear and tassel. The enrichment of DMRs in the differential OCRs is not significantly different from the DMRs in randomly selected OCRs (same number with differential OCRs as controls, repeat 500 times). We therefore speculate that the DMRs are not correlated with dynamic chromatin accessibilities.
We further investigated the overlapping fraction of the DMRs with DEGs and total genes, we found the DMRs are overlapping with 34% of all the DEGs (actual) and 30% of total maize genes (control), which are similar. So, we speculate that the connection between DMRs and DEGs is not obvious between tassel and ear datasets.*

Reviewer #2: This paper provides a high quality, rich resource that will be a key enabling resource for the maize genetics community. It provides a significant advance over previous studies and will be used by others to further examine the complex regulation of genes that specify the ear and tassel in maize. It also nicely ties to previous results on specific transcription factors that function in the ear and tassel. The paper is of broad interest to the biology community and especially the maize community.
Comment 2.1 The methods are appropriate, although, other than the ATAC-seq data, I am not sure if all of the datasets were replicated? This should be clearly stated in the materials and methods. and/or the results section.

*Response 2.1 We fully agree with the Reviewer about the importance of clarifying replicate information. Our RNA-seq datasets have 3 replicates, and ATAC-seq, ChIP-seq and Hi-C datasets have 2 replicates for each tissue. Since the appropriate internal control was setup in BS-seq, our DNA methylation data has one replicate with sufficient sequencing depth (30X). We provide the replicate information in the Methods section in the revised manuscript (line 541-543).*

Comment 2.2 It seems like a single cell-based approach would have resulted in an even more robust dataset and would be more specific with regards to the various OCR, chromatin marks, etc. and their impact on gene regulation in both the ear and tassel.

*Response 2.2 The Reviewer raised an interesting question. We agree that the single cell-based approaches mapping OCRs and various chromatin marks have been applied in animals. However, to the best of our knowledge, no single-cell genome-wide OCRs or chromatin marks study was reported so far in plants. A possible reason might be the difficulty to prepare high quality single plant cells. A few studies reported the scRNA-seq from Arabidopsis root tips (Ryu et al., 2019; Shulse et al., 2019; Zhang et al., 2019), but may be limited by the sparsity of data that is inherent to current single-cell assays. It usually exhibits the signals by aggregating data from many single cells. Anyway, we agree that it is absolutely worth exploring the impacts of various OCRs and chromatin marks on transcriptional regulation in both the ear and tassel at single cell level with robust methods in the future.*

Comment 2.3 On page 6 line 146 it says "significantly more active than those with repressive H3K27me3 or lacking modifications". One of the genes associated with H3K27me3 also had other marks (H3K4me3, H3K9me3) and exhibited high expression. I think this should be clarified.

*Response 2.3 We apologize for the confusion raised since we made a mistake during data sorting and prepared the plot (Fig 1f and Figure S2b) using improperly sorted data (also see comments to Reviewer 1). In Fig 1f and Figure S2b, the expression values were previously plotted as 10, 1, 2, 3…9, whereas the correct order should be 1, 2, 3…10. The revised figures show that genes with high H3K27me3 do have a low expression level, and other conclusions are not affected.*

Comment 2.4 There are several places in the text where it is stated that the comparison was "significant" or something like "exhibited more active chromatin and active H3K4me3 ... " (page 7 line 174-175) and gene expression differences (page 7 178-179). A statistical test should be shown in these cases.

*Response 2.4 Thanks for your suggestion, we have added the statistical tests in lines 177, 183-185, 298, 311 and their related figures in Fig 2b-d, 2f, 2g, Fig 4i, 4j, 4n and Fig S8g, h.*

Comment 2.5 On page 11-12 Lines 283-284 a two-tailed test is performed on randomly selected gene pairs, I think an FDR should be shown here.

*Response 2.5 Here, we aim to compare the expression correlation between gene pairs with chromatin loops and randomly selected gene pairs tested by two-tailed test. In statistics, the false discovery rate (FDR) is a method of conceptualizing the rate of type I errors in null hypothesis test when conducting multiple comparisons. The comparison here is only performed once (looped gene pairs vs randomly selected pairs), therefore we only showed the p-value.*

Comment 2.6 In figure 2 and in the corresponding results section, why wasn't the H3K9me3 data described?

*Response 2.6 We didn’t generate the H3K9me3 data in the paper, so we guess you mean H3K9ac data. Originally, we analyzed the H3K9ac data, but found it was not obviously correlated with the differential expression of genes in ear and tassel. This might be due to the very broad H3K9ac peaks called in our study. In addition, H3K9ac is mainly associated with circadian gene expression (Baerenfaller et al., 2016). Therefore, we didn’t describe the H3K9ac data in Figure 2.*

Comment 2.7 In the gene co-expression network (Figure 3h) the authors used 65 RNA-seq datasets from B73. Since these datasets are not from just the ear and tassel, it may be a stretch to say they interact in the tassel. In addition, in the figure 3 legend it says that these are potentially regulated genes. I am unclear if these genes were shown to regulate it other. Without further data the only that can be said is that the genes are co-expressed.

*Response 2.7 In the Figure 3h, we performed regulatory network analysis of transcription factors by applying maize publicly available ChIP-seq data. This part did not involve the 65 RNA-seq datasets. The co-expression analysis using the 65 RNA-seq datasets from B73 was performed for the gene pairs with chromatin loops (please refer to lines 289-292 and Fig 4h).

References cited in the Response to Referees
Baerenfaller, K., Shu, H., Hirsch‐Hoffmann, M., Fütterer, J., Opitz, L., Rehrauer, H., Hennig, L., and Gruissem, W. (2016). Diurnal changes in the histone H3 signature H3K9ac|H3K27ac|H3S28p are associated with diurnal gene expression in Arabidopsis. Plant, Cell & Environment 39:2557–2569.
Clark, R. M., Wagler, T. N., Quijada, P., and Doebley, J. (2006). A distant upstream enhancer at the maize domestication gene tb1 has pleiotropic effects on plant and inflorescent architecture. Nat Genet 38:594–597.
Galli, M., Khakhar, A., Lu, Z., Chen, Z., Sen, S., Joshi, T., Nemhauser, J. L., Schmitz, R. J., and Gallavotti, A. (2018). The DNA binding landscape of the maize AUXIN RESPONSE FACTOR family. Nature Communications 9:1–14.
Garg, R., Narayana Chevala, V., Shankar, R., and Jain, M. (2015). Divergent DNA methylation patterns associated with gene expression in rice cultivars with contrasting drought and salinity stress response. Scientific Reports 5:1–16.
O’Malley, R. C., Huang, S.-S. C., Song, L., Lewsey, M. G., Bartlett, A., Nery, J. R., Galli, M., Gallavotti, A., and Ecker, J. R. (2016). Cistrome and Epicistrome Features Shape the Regulatory DNA Landscape. Cell 165:1280–1292.
Ryu, K. H., Huang, L., Kang, H. M., and Schiefelbein, J. (2019). Single-Cell RNA Sequencing Resolves Molecular Relationships Among Individual Plant Cells. Plant Physiology 179:1444–1456.
Shulse, C. N., Cole, B. J., Ciobanu, D., Lin, J., Yoshinaga, Y., Gouran, M., Turco, G. M., Zhu, Y., O’Malley, R. C., Brady, S. M., et al. (2019). High-Throughput Single-Cell Transcriptome Profiling of Plant Cell Types. Cell Reports 27:2241-2247.e4.
Yan, W., Chen, D., Schumacher, J., Durantini, D., Engelhorn, J., Chen, M., Carles, C. C., and Kaufmann, K. (2019). Dynamic control of enhancer activity drives stage-specific gene expression during flower morphogenesis. Nat Commun 10:1–16.
Zhang, T.-Q., Xu, Z.-G., Shang, G.-D., and Wang, J.-W. (2019). A Single-Cell RNA Sequencing Profiles the Developmental Landscape of Arabidopsis Root. Molecular Plant 12:648–660.
Zhu, B., Zhang, W., Zhang, T., Liu, B., and Jiang, J. (2015). Genome-Wide Prediction and Validation of Intergenic Enhancers in Arabidopsis Using Open Chromatin Signatures. The Plant Cell 27:2415–2426.*

**Second round of review**

**Reviewer 1**

The revised manuscript has been significantly improved. It is a pity that the authors could not provide additional experimental data to show how TF/genome interactions are connected to other local chromatin features (e.g. chromatin accessibility) in distinct tissues. Therefore, I re-assessed figures and texts in which association analyses about ChIP-seq were presented.

Frankly, I agree that it is reasonable to speculate that a TF would bind to an open chromatin region in one tissue, when it has been experimentally shown that this TF can bind to the same region in another tissue. However, to me, the next-level speculation, which is presented as a model in Fig3g, is rather misleading. Please note that this model is based on an ASSUMPTION that all the TF-chromatin interactions occur, and an additional ASSUMPTION that these interactions occur simultaneously. For now, the authors should list other scenarios, for instance, sequential TF-DNA binding, or the contribution of tissue heterogeneity, which also fit the data patterns.

In addition, please revise (or remove) Fig3b,3d, and 3e:
Fig 3b: it presents comparisons between q-value sorted ATAC-seq peaks and ChIP-seq signals. For KN1 and FEA4, please specify tissue type; for TB1, please remove it. (the TB1 ChIP data is from vegetative tiller buds, it does not work out by sorting these ChIP peaks according to q-values of ATAC-seq peaks in ear/tassel)
Figs 3d and 3e, I suggest removing TB1. (those numbers belonging to TB1 were based on speculative data filtering)

**Reviewer 2**

The authors have adequately addressed my comments.

**Authors Response**

**Point-by-point responses to the reviewers’ comments:**

Reviewer #1: The revised manuscript has been significantly improved. It is a pity that the authors could not provide additional experimental data to show how TF/genome interactions are connected to other local chromatin features (e.g. chromatin accessibility) in distinct tissues. Therefore, I re-assessed figures and texts in which association analyses about ChIP-seq were presented.

Frankly, I agree that it is reasonable to speculate that a TF would bind to an open chromatin region in one tissue, when it has been experimentally shown that this TF can bind to the same region in another tissue. However, to me, the next-level speculation, which is presented as a model in Fig3g, is rather misleading. Please note that this model is based on an ASSUMPTION that all the TF-chromatin interactions occur, and an additional ASSUMPTION that these interactions occur simultaneously. For now, the authors should list other scenarios, for instance, sequential TF-DNA binding, or the contribution of tissue heterogeneity, which also fit the data patterns.

*Response. We highly appreciate your insightful comments. Based on your description, we think you meant the model in Fig. 5g, instead of Fig 3g that need to be revised. The proposed model for the regulation of TB1 aims to show that the distal cis regulatory element (CRE) of TB1 is more open with less H3K27me3 modification in ear than in tassel, which might provide a better accessibility for multiple TFs. We agree with you that TF-chromatin interactions could have multiple scenarios, such as sequential TF/genome interactions, simultaneous interactions and also differential interactions between tissues. Following your suggestions, we have revised the model for better clarity (see details below) by separating multiple TFs to show a comprehensive TF-DNA binding patterns. Particularly, the different TFs are represented by the hexagons with different colors, some of which might interact each other and bind to the CRE as a complex. The sequential TF-DNA binding is shown by the arrows and the distance of TFs from the CRE. The different TF-DNA binding patterns between tassel and ear are illustrated by the differences in the number and types of TFs.*

In addition, please revise (or remove) Fig3b,3d, and 3e:

Fig 3b: it presents comparisons between q-value sorted ATAC-seq peaks and ChIP-seq signals. For KN1 and FEA4, please specify tissue type; for TB1, please remove it. (the TB1 ChIP data is from vegetative tiller buds, it does not work out by sorting these ChIP peaks according to q-values of ATAC-seq peaks in ear/tassel).

Figs 3d and 3e, I suggest removing TB1. (those numbers belonging to TB1 were based on speculative data filtering).

*Response. We followed your suggestion and removed the ChIP-seq data of TB1 in the revised Fig 3b, 3d, 3e and specified the tissue from which the ChIP-seq data for KN1 and FEA4 were generated. Besides, the corresponding main text was also changed in the revised manuscript.*

Reviewer #2: The authors have adequately addressed my comments.

*Response Thank you very much for your positive comments.*

**Third round of review**

**Reviewer 1**

The revised manuscript has satisfactorily addressed all my concerns. The revision is well done, thanks!
